# Estimating the impact of cancer diagnosis on life expectancy by stage at diagnosis: population-based estimates for a range of cancer sites in England

**DOI:** 10.1136/bmjonc-2025-000999

**Published:** 2026-04-30

**Authors:** Rachael Stannard, Paul C Lambert, Therese M-L Andersson, Sam Khan, Georgios Lyratzopoulos, Elisavet Syriopoulou, Mark J Rutherford

**Affiliations:** 1 Division of Public Health and Epidemiology, University of Leicester, Leicester, UK; 2 Department of Medical Epidemiology and Biostatistics, Karolinska Institutet, Stockholm, Sweden; 3 Cancer Registry of Norway, Norwegian Institute of Public Health, Oslo, Norway; 4 Leicester Cancer Research Centre, Leicester Royal Infirmary, University of Leicester, Leicester, UK; 5 Epidemiology of Cancer Healthcare & Outcomes (ECHO), Department of Behavioural Science and Health, Institute of Epidemiology & Health Care (IEHC), University College London, London, UK

**Keywords:** Biostatistics, Mortality, Survival Analysis

## Abstract

**Objective:**

Unlike survival measures, life expectancy readily illustrates the burden of cancer on society and the average impact on individuals diagnosed with cancer. Cancer stage at diagnosis is a key prognostic factor and hence it is important to obtain stage-specific life expectancy estimates. However, completeness of recording for cancer stage at diagnosis is often historically poor in cancer registries. Therefore, it can be challenging to obtain the long-term stage-specific survival estimates required for estimating stage-specific life expectancy. We provide the first stage-specific life expectancy estimates using whole population data in England.

**Methods and analysis:**

Multiple imputation was used to impute values of cancer stage at diagnosis for patients with missing stage at diagnosis information. The simultaneous application of period analysis to obtain up-to-date estimates restricts the contribution of patients with historical diagnoses and hence improves the overall completeness of stage at diagnosis information. For each of the 10 cancer sites in this study, we fit a flexible parametric excess hazard model for each cancer stage on the cumulative excess hazard scale and estimated stage-specific life expectancy using the relative survival framework.

**Results:**

The differences in stage-specific and sex-specific life expectancy were evaluated from 40 to 90 years of age. Colorectal cancer, prostate cancer and bladder cancer yield much lower estimates of life expectancy for patients diagnosed with stage IV cancer compared with stages I–III. For example, female patients diagnosed with stage IV colorectal cancer at 70 years of age have a life expectancy of 72.3 years, while those with stages I–III can expect to live beyond 82.0 years. The remaining cancer sites yield approximately equal reductions in life expectancy with each increase in stage at diagnosis from I to IV.

**Conclusion:**

We offer the first stage-specific life expectancy estimates for a range of cancer sites in England using data from the National Cancer Registration and Analysis Service, with follow-up until February 2020. Estimates of stage-specific life expectancy provide a real-world, intuitive metric to evaluate the impact of cancer stage at diagnosis on prognosis up to a lifetime horizon. Stage-specific life expectancy estimates also provide key information regarding the potential benefits of early diagnosis initiatives in terms of gains in life years.

**WHAT IS ALREADY KNOWN ON THIS TOPIC**

- Stage-specific life expectancy is an easy-to-understand metric, which can be directly compared with general population life expectancy estimates to understand the impact of cancer.
- Cancer stage at diagnosis is an important prognostic factor; however, completeness of recording of cancer stage at diagnosis can be historically poor in cancer registration data resources.

**WHAT THIS STUDY ADDS**

- This study offers the first stage-specific life expectancy estimates for a range of cancer sites in England, derived from whole-population data, using recently developed methods.

**HOW THIS STUDY MIGHT AFFECT RESEARCH, PRACTICE OR POLICY**

- This work has highlighted target groups for the improvement of incidence and mortality rates and facilitates the continued monitoring of trends over time.
- Comparisons across stage-specific estimates for a given age at diagnosis can motivate the life expectancy gains that could be achieved through early diagnosis initiatives.

## Introduction

 Monitoring the progress towards reducing the burden of cancer is critical for the planning and delivery of services and in the evaluation of diagnostics and treatments.^1^ In addition to temporal comparisons, comparisons are often made between populations and risk factor groups to explore inequalities in cancer outcomes.^1^ All-cause survival and related measures, even if evaluated after long follow-up, are associated with a specific point in time after diagnosis and do not capture the lifetime impact of cancer on survival outcomes. In contrast, life expectancy provides a tangible metric to measure the overall burden of cancer among affected patients.^2^ Life expectancy metrics can be adjusted to reflect quality of life, scaled by incidence statistics to understand population burden,^3^ and are compatible with cost-effectiveness analyses.

Stage-specific life expectancy estimates can inform policy by improving planning and resource allocation, supporting benchmarking across services, strengthening early-diagnosis initiatives, highlighting inequalities in outcomes and enhancing cost-effectiveness analyses. Clinically, stage-specific information enables more personalised prognostic discussions and tailoring of care. Since life expectancy is an intuitive metric, it can improve communication between clinicians and patients. Together, these benefits support both system-level improvement and more informed individual decision-making.

Cancer stage at diagnosis is an important prognostic factor and hence stage-specific estimates are of great interest. However, completeness of recording of cancer stage at diagnosis can be historically poor in cancer registration data resources,^4^ making it challenging to provide the long-term stage-specific survival estimates required for estimating stage-specific life expectancy. There are many reasons for incomplete stage at diagnosis. Stage at diagnosis may never have been determined or not recorded after the fact. Stage at diagnosis may not be recorded due to a coding error. Patients whose stage at diagnosis was never determined often share characteristics such as having more advanced disease, being older or having multiple comorbidities.^5^ Many cancer registries have improved stage completeness in recent years. EUROCARE 4 showed cancer outcomes in England were less favourable than in the best performing European countries.^6^ A 5-year cancer plan, the Cancer Reform Strategy for England, was published in 2007, noting the need for higher completeness of stage recording.^7^ Following this, the National Awareness and Early Diagnosis Initiative was launched in 2008 by the Department of Health and Cancer Research UK.^8^ In 2011, Improving Outcomes: A Strategy for Cancer^9^ was launched. This initiative commented on the need to further improve the completeness of the recording of stage at diagnosis. By 2012, stage completeness had greatly improved.^4^ For 24 selected cancer sites in England, cancer stage information was available for 85.3% of patients diagnosed in 2013–2017.^10^ In England, it has been possible to provide short-term stage-specific survival estimates for the whole population. An annual report detailing cancer survival in England published by the Office for National Statistics in 2019 was the first of its kind to report 5-year stage-specific net survival.^11^ Other studies have restricted their analysis to the East of England, where staging information was more complete.^12 13^ However, due to the incomplete historical stage information at a population level, it has not been possible to use whole population data to provide long-term survival or life expectancy separately by stage to date.

We previously showed that combining period analysis with multiple imputation yields long-term stage-specific survival estimates that are robust to imputed historical stage information when short-term prognosis drives survival differences.^14^ Here, we extend this framework from relative survival to life expectancy, presenting the first stage-specific life expectancy estimates for multiple cancer sites in England. We provide contemporary, sex-specific estimates for 10 cancers, offering a comprehensive measure of cancer’s lifetime impact on survival and illustrating how stage at diagnosis, age and sex influence differences from general-population life expectancy. We describe the data and statistical methods used to address missing stage information and generate these estimates.

## Methods

### Data

Data from the English Cancer Registry (National Disease Registration Service (NDRS)) were extracted for patients diagnosed with bladder (International Classification of Diseases, Tenth Revision (ICD-10) C67), breast (ICD-10 C50), cervix uteri (ICD-10 C53), colorectal (ICD-10 C18-C20), Hodgkin lymphoma (ICD-10 C81), lung (ICD-10 C33-C34), melanoma (ICD-10 C43), ovary (ICD-10 C56-C57), prostate (ICD-10 C61) and stomach (ICD-10 C16) cancer between 2006 and 2020, with follow-up until the end of February 2020. ICD-10 codes C56 and C57 include cancers of both the ovary and the fallopian tube. Death certificate-only cases and male patients with breast cancer were excluded. Each cancer site was analysed separately.

A period analysis approach was performed to estimate up-to-date life expectancy, while making use of the data available.^15 16^ A standard analysis would include the entire follow-up recorded for each patient, regardless of year of diagnosis. A period analysis ensures only patients diagnosed recently can impact the short-term estimates, while still using the long-term follow-up of patients diagnosed in the preceding years. The selected period window was 1 January 2017 until 29 February 2020, with follow-up restricted to 10 years. A schematic can be found in online supplemental appendix A, figure A1. Restricting to 10 years of follow-up aids model stability, and the completeness of stage is better in recent calendar years. The performance of life expectancy extrapolation using the relative survival framework with 10 years of follow-up has been evaluated, and deemed to be sufficient.^17 18^

Age at diagnosis is recorded in years. The age information received from the NDRS assumes all patients are diagnosed on their birthday, and are therefore on average 0.5 years younger than the truth. Hence, the age of all patients was increased by 0.5 years. Only patients aged 18 years or older were included. Sex is recorded as male or female, self-stated at diagnosis. Male patients diagnosed with breast cancer were excluded. Cancer stage at diagnosis is recorded as stage I, II, III or IV. Cancer stage is derived using a combination of T, N and M stage by the NDRS.^19^ T, N and M stage denote the local size of the tumour, growth of the tumour through local tissues and involvement of lymph nodes and metastases to other organs, respectively. T, N and M staging is performed through a combination of imaging and pathology. Patients diagnosed with Hodgkin lymphoma are primarily staged using the Ann Arbor staging system. Patients diagnosed with cervical cancer and ovarian cancer are primarily staged using the International Federation of Gynecology and Obstetrics (FIGO) staging system. Patients diagnosed with other cancers are primarily staged using the UICC 8 staging system, with some patients staged using Union for International Cancer Control (UICC) 7. Other staging systems are also used and full details are given in online supplemental appendix B, table A1. Deprivation is recorded from 1 (most deprived) to 5 (least deprived) as provided by the NDRS at small area level derived from the Index of Multiple Deprivation (IMD) domain in 2019 with quintiles weighted equally by the number of lower layer super output areas.

### Life expectancy

The life expectancy for covariate pattern *j* is estimated as: $LE_{j}=\int_{0}^{\infty }\;S^{*}\left(u;z_{j}^{\prime }\right)R\left(u;z_{j}\right)\;du$, where *z*_*j*_ denotes the covariates associated with relative survival. The expected survival at time t, S*(t), is estimated using life tables stratified by covariates *z_j_’*. *R(t)* denotes relative survival at time *t* and is obtained from flexible parametric relative survival models.

Life expectancy metrics are estimated using the relative survival approach^17^ to compare differences in stage-specific and sex-specific life expectancy from 40 to 90 years of age. Life expectancy estimates are presented from 30 to 90 years for cervical cancer and Hodgkin lymphoma due to the younger age profile of patients. For prostate cancer, life expectancy estimates are presented from 50 to 90 years since there were few patients diagnosed at younger ages.

The estimation process of life expectancy requires the all-cause survival function to reach zero. However, the all-cause survival function rarely reaches zero within the timespan of data available, particularly for covariate profiles with better prognosis, and hence it is typical to extrapolate the survival function. It has previously been shown that extrapolation of the all-cause survival estimate is more reliable by extrapolating relative survival estimates before converting to all-cause survival. This approach uses population mortality information from life tables that appropriately accounts for the effect of ageing on other-cause mortality as part of the extrapolation process. Therefore, although life expectancy is an all-cause measure, we use the relative survival framework to reliably extrapolate all-cause survival.^17 18^ Since life expectancy is an all-cause measure, when comparing life expectancy across subgroups of patients with cancer, it is important to recognise that observed differences may stem from variations in other-cause mortality, differences in cancer-specific mortality or both.

Life expectancy estimates were calculated using life tables obtained from the Human Mortality Database (HMD).^20^ The life table is stratified by calendar year, sex and age. The estimates of life expectancy from the 50 data sets obtained by multiple imputation were then combined using Rubin’s rules.^21^

### Multiple imputation

Missing cancer stage at diagnosis was imputed under a multinomial logistic model with 50 imputed datasets.^22^ Stage at diagnosis was only imputed for individuals who contribute person-time to the period window. The variables selected for the imputation model are: sex, age, year of diagnosis, deprivation, an interaction term between sex and age and interaction terms between calendar year and age, sex and deprivation. The imputation model also includes terms for the event indicator, the Nelson-Aalen estimate of cumulative hazard and further interactions and transformations to account for the fact that the effect of covariates on the excess hazard are time-dependent.^23^ Age was modelled continuously with splines using the Stata command gensplines.^24^ Further details are given in online supplemental appendix C.

### Flexible parametric survival model

When using cancer registry-based data, it is common to observe deaths due to causes other than the cancer under study. The relative survival framework provides an approach to account for competing risks, without the need for cause of death information, as required by the cause-specific approach, which is often unavailable or unreliable.

Flexible parametric models were fitted on the cumulative excess hazard scale.^25^ A separate model was defined for each cancer stage and site to enable covariate effects to vary by stage at diagnosis, without needing to impose assumptions regarding stage as this is the primary covariate of interest. Flexible parametric models were chosen over other methods, such as Cox proportional hazards models, due to their capacity to flexibly capture complex relationships, and for the crucial extrapolation required to estimate life expectancy.

The selected flexible parametric survival models included age and sex as main covariates, an interaction between age and sex and time-dependent effects of age and sex. Age was modelled using natural splines and winsorised to provide more stability in the tails of the data.^26^ Time-dependent effects of age were modelled using natural splines. The analysis models were fitted using the Stata command stpm3.^27^ Further details are given in online supplemental appendix C.

To ensure model convergence across all of the cancer site and stages, and for each of the 50 imputations, a set of candidate models were defined, with each candidate model decreasing in complexity from the target model.^28^ If the target model failed to converge, a simpler model was attempted. The full list of candidate models and selection frequency is given in online supplemental appendix D, tables A2 and A3.

Flexible parametric excess hazard models require appropriate expected population mortality rates to separate the excess hazard associated with cancer from the all-cause hazard. The life table containing the mortality rates was obtained from the NDRS.^29^ The life table is stratified by calendar year, sex, age, IMD and government office region (GOR). IMD quintile is coded from 1 (most deprived) to 5 (least deprived). This finer degree of stratification by IMD quintile and GOR is beneficial when fitting the excess hazard model. It should be noted that we do not include GOR and deprivation as covariates in our excess mortality model, despite them being included in the corresponding population mortality files. We therefore estimate excess mortality rates that depend only on age, sex and stage, but appropriately match individuals based on all factors in the life table. We then use the HMD life table not stratified by IMD and GOR to obtain life expectancy to simplify the prediction process and give a direct and easy approach to comparing across cancer sites that potentially differ, such as by deprivation.^30^

Survival of patients diagnosed with stage I and stage II prostate cancer is typically very good, sometimes greater than the population average, and hence fitting excess hazard models can be challenging.^31^ Life expectancy estimates are not presented for patients with stage I and stage II prostate cancer due to convergence and prediction difficulties, but these would be expected to be close to life expectancy in the general population.

### Non-parametric validation

Estimates of 10-year relative survival obtained from the parametric models were compared with the non-parametric Pohar-Perme estimator^32 33^ to validate the flexible parametric model estimates. This is to ensure any differences in sex-specific and stage-specific life expectancy are not due to model assumptions. The estimates are obtained within the five International Cancer Survival Standard age groups (<45, 45–54, 55–64, 65–74, 75+ years)^34^ and are given in online supplemental appendix E, figures A2–A11.

All analyses were conducted using statistical software Stata V.18,^35^ and the code is available at https://github.com/RStannard1/Stage-Specific-Life-Expectancy-2025.

## Results

Table 1 presents the baseline characteristics for patients diagnosed with breast, colorectal, lung, melanoma and prostate cancer between 1 January 2017 and 31 December 2019. Online supplemental appendix F, table A4 gives the corresponding baseline characteristics for bladder, cervical, Hodgkin lymphoma, ovarian and stomach cancer. The total number of cases is given for each cancer site. Prostate cancer has the greatest incidence with 139 249 cases (average of 46 416 cases per year), closely followed by breast cancer with 129 226 cases (average of 43 075 cases per year). Hodgkin lymphoma has the smallest incidence with 4645 cases (average of 1548 cases per year), followed by cervical cancer with 7940 cases (average of 2647 cases per year). The age distribution of patients is given in years as median (25th percentile, 75th percentile) by cancer site. Bladder cancer has the highest median age at 76 (69, 83), while cervical cancer has the lowest median age at 44 (34, 59). The number and percentage of male and female patients are given by site. Cervical, ovarian and prostate cancer are single-sex sites from data extraction. The number and percentage of patients diagnosed at each stage are given by site. The percentages for stages I–IV sum to 100 and a separate percentage is given for patients with missing stage at diagnosis with respect to the total number of patients. Stomach cancer has the greatest proportion of missing data at 24%, followed by bladder cancer (19%), cervical cancer (17%) and ovarian cancer (17%). The number and percentage of patients in each deprivation quintile are given separately by cancer site. Lung cancer exhibits the greatest proportion of patients in the most deprived quintile compared with the other sites, and has the smallest proportion of patients in the least deprived quintile. Prostate cancer has the smallest proportion of patients in the most deprived quintile compared with other sites, while also having the greatest proportion of patients in the least deprived quintile.

**Table 1:** Baseline characteristics for patients diagnosed between 2017 and 2019

		Cancer site				
		Breast	Colorectal	Lung	Melanoma	Prostate
Total (n)		129 226	104 391	115 037	40 995	139 249
Age (years)*		63 (52, 73)	72 (63, 81)	73 (66, 80)	66 (52, 76)	71 (64, 77)
Sex†	Male	0 (0)	58 188 (56)	60 073 (52)	20 812 (51)	139 249 (100)
	Female	129 226 (100)	46 203 (44)	54 964 (48)	20 183 (49)	0 (0)
Stage†‡	I	50 766 (44)	16 964 (19)	21 898 (21)	25 563 (68)	45 218 (38)
	II	48 664 (42)	23 229 (25)	8490 (8)	7823 (21)	19 277 (16)
	III	10 903 (9)	28 145 (31)	22 514 (21)	2866 (8)	31 040 (26)
	IV	5986 (5)	23 199 (25)	52 943 (50)	1101 (3)	24 609 (20)
	Unknown	12 907 (10)	12 854 (12)	9192 (8)	3642 (9)	19 105 (14)
Deprivation†§	1 (Most)	20 069 (16)	17 084 (16)	30 209 (26)	4448 (11)	18 216 (13)
	2	23 358 (18)	18 999 (18)	24 506 (21)	6365 (16)	23 971 (17)
	3	27 040 (21)	21 898 (21)	22 506 (20)	8549 (21)	29 568 (21)
	4	28 921 (22)	23 095 (22)	20 585 (18)	10 145 (25)	32 788 (24)
	5 (Least)	29 838 (23)	23 315 (22)	17 231 (15)	11 488 (28)	34 706 (25)

* Are given as median (25th percentile, 75th percentile).

† Are given as n (%).

‡ Percentages for stages I–IV sum to 100, with separate per cent given for unknown stage.

§ Deprivation graded from 1 (most deprived) to 5 (least deprived).

**Figure 1:**
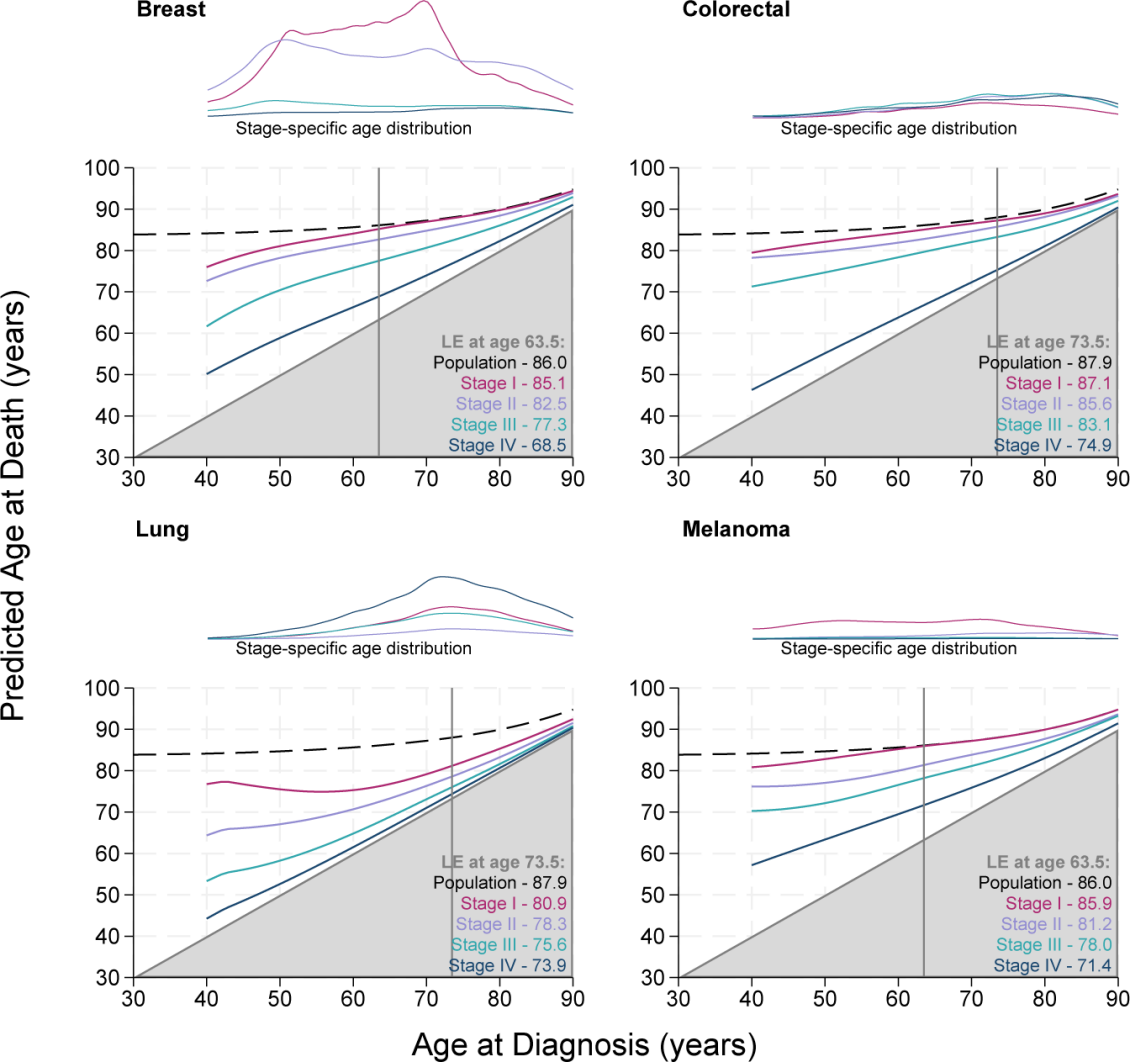
Stage-specific life expectancy (LE) estimates for female patients aged 40–90 years diagnosed with breast cancer, colorectal cancer, lung cancer and melanoma. Dashed black lines display the LE of the general population (cancer free). Vertical grey line marks the median age at diagnosis, with associated LE estimates given for the general population and stages I–IV in lower right corners. Line plots illustrate the stage-specific age distribution of patients diagnosed between 2017 and 2019. The lower right region represents survival <0 years from diagnosis and is therefore shaded grey. The age distributions are comparable within this panel as the plots are on a common scale, but not across other subpanels in figures2^4^. CIs can be found in online supplemental appendix G, tables A5 and A6.

Figures1^4^ display the stage-specific life expectancy (predicted age at death) separately by stage I–IV, with a corresponding plot illustrating the age distribution at diagnosis for individuals diagnosed between 2017 and 2019, for each stage group I–IV separately. The life expectancy for the general population is also given in the form of a black dashed line. Hence, estimates that are closer to this line and further away from the grey shaded area indicate a better prognosis. The lower right region represents survival <0 years from diagnosis and is hence shaded grey. Here, the y-axis gives the mean age at death, rather than estimated life years remaining, as is often seen. Online supplemental appendix G, tables A5 and A6 display these life expectancy estimates with 95% CIs for patients diagnosed at 40, 50, 60, 70 and 80 years of age. The figures and tables in this study detail life expectancy as predicted age at death. Remaining life expectancy can be obtained simply by subtracting age at diagnosis from the predicted age at death.

**Figure 2:**
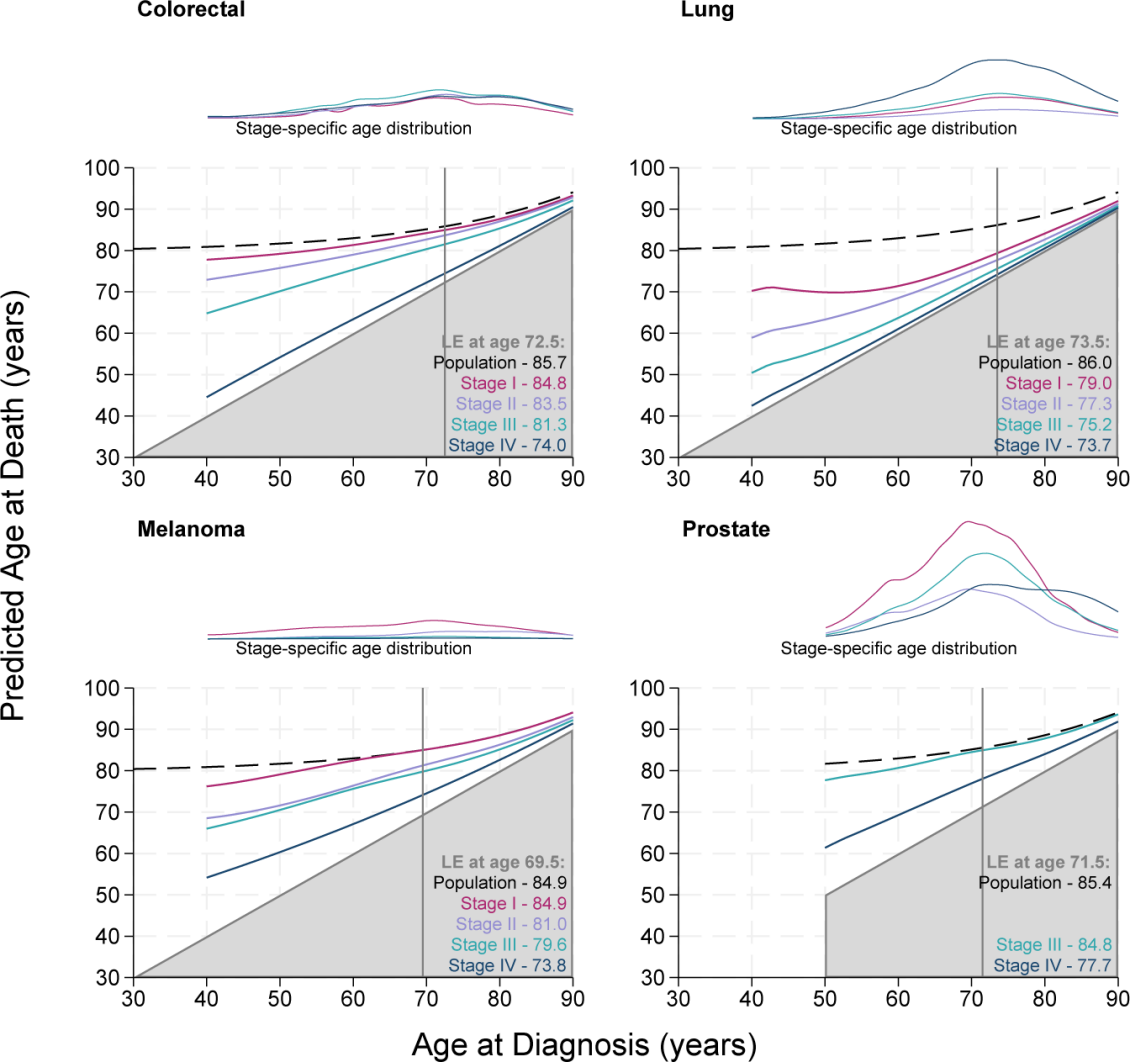
Stage-specific life expectancy (LE) estimates for male patients aged 40–90 years diagnosed with colorectal cancer, lung cancer, melanoma and prostate cancer. Dashed black lines display the LE of the general population (cancer free). Vertical grey line marks the median age at diagnosis, with associated LE estimates given for the general population and stages I–IV in lower right corners. Line plots illustrate the stage-specific age distribution of patients diagnosed in 2017–2019. The lower right region represents survival of <0 years from diagnosis and is therefore shaded grey. The age distributions are comparable within this panel as the plots are on a common scale, but not across other subpanels in figures1, 3  4. CIs can be found in online supplemental appendix G, tables A5 and A6.

**Figure 3:**
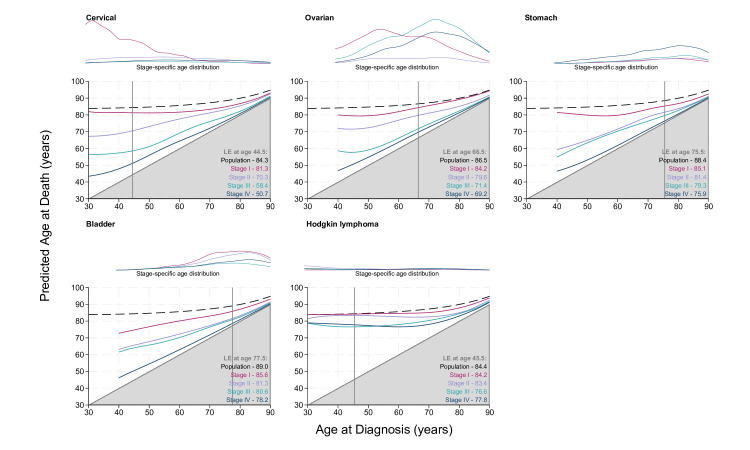
Stage-specific life expectancy (LE) estimates for female patients aged 40–90 years diagnosed with cervical cancer (aged 30–90 years), ovarian cancer, stomach cancer, bladder cancer and Hodgkin lymphoma (aged 30–90 years). Dashed black lines display the LE of the general population (cancer free). Vertical grey line marks the median age at diagnosis, with associated LE estimates given for the general population and stages I–IV in lower right corners. Line plots illustrate the stage-specific age distribution of patients diagnosed in 2017–2019. The lower right region represents survival <0 years from diagnosis and is therefore shaded grey. The age distributions are comparable within this panel as the plots are on a common scale, but not across other subpanels in figures1, 2  4. CIs can be found in online supplemental appendix G, tables A5 and A6.

**Figure 4:**
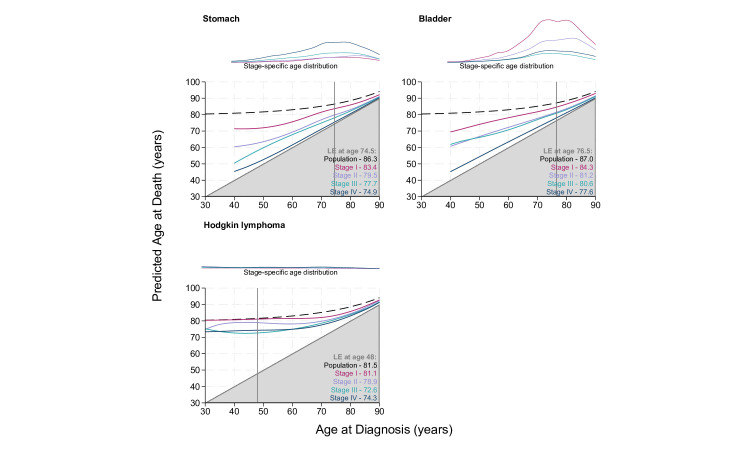
Stage-specific life expectancy (LE) estimates for male patients aged 40–90 years diagnosed with stomach cancer, bladder cancer and Hodgkin lymphoma (aged 30–90 years). Dashed black lines display the LE of the general population (cancer free). Vertical grey line marks the median age at diagnosis, with associated LE estimates given for the general population and stages I–IV in lower right corners. Line plots illustrate the stage-specific age distribution of patients diagnosed in 2017–2019. The lower right region represents survival <0 years from diagnosis and is therefore shaded grey. The age distributions are comparable within this panel as the plots are on a common scale, but not across other subpanels in figures1^3^. CIs can be found in online supplemental appendix G, tables A5 and A6.

Figure 1 displays the stage-specific life expectancy for female patients diagnosed with breast, colorectal, lung and melanoma cancer from age 40 to 90 years. Patients diagnosed with stage I breast cancer at the median age of 63.5 years live, on average, to 85.1 years, compared with 86.0 years in the general population. Those diagnosed at the median age of 63.5 years with stage II live, on average, to 82.5 years, stage III 77.3 years and stage IV 68.5 years. The stage-specific age distribution plot indicates much greater incidence at stages I and II than stages III and IV. Breast cancer, lung cancer and melanoma show similar trends of decreased life expectancy with each stage I–IV. Colorectal cancer displays a deviation from this trend where although still ordinal, stages I–III are similar and stage IV yields a significantly shorter life expectancy. The life expectancy estimates for patients diagnosed with stage IV colorectal cancer and stage IV lung cancer remain very close to the grey area for all ages, indicating very poor prognosis. The stage-specific age distribution plot indicates much greater incidence of lung cancer at stage IV than stages I–III across all ages. The estimated life expectancy of patients diagnosed with stage I melanoma is very similar to that of the general population across all ages.

The stage-specific age distribution plots indicate a greater incidence of breast cancer and melanoma at stage I than stages II–IV across all ages, whereas stage IV has the greater incidence among patients with lung cancer.

Stage-specific age distribution plots are given in online supplemental appendix H, figure A12.

Figure 2 gives the stage-specific life expectancy for male patients diagnosed with colorectal, lung and melanoma cancer from age 40 to 90 years. Similar patterns for incidence and life expectancy are seen here compared with the results for female patients with lung cancer, colorectal cancer and melanoma in figure 1. While the estimated life expectancy of male and female patients diagnosed with stage IV colorectal cancer remains similar, the estimated life expectancy of male patients diagnosed with stages I–III is lower than that of female patients. This difference is largely driven by differences in non-cancer mortality, illustrated by the decline in population life expectancy in black compared with the corresponding population life expectancy estimate given in figure 1. Similarly, estimates of life expectancy are notably lower for male patients with lung cancer compared with female patients. Although these differences in life expectancy by sex are partly driven by differences in non-cancer mortality, sex-based differences in lung cancer outcomes are widely reported.^36 37^ These differences may be partly due to differences in natural history, treatment response and in anticancer treatment after diagnosis: men are less likely to receive surgery, and this has been linked to the average age at diagnosis, smoking status and the number of comorbidities.^38^ Life expectancy is dependent on both cancer and other-cause mortality, and we do not recommend making direct comparisons across sex. The proximity between life expectancy estimates for patients diagnosed with stage II and stage III melanoma indicates a lesser impact of stage at diagnosis here compared with stage I to stage II and stage III to stage IV.

Figure 2 also shows the stage-specific life expectancy for male patients diagnosed with prostate cancer. Life expectancy estimates for patients diagnosed with prostate cancer are given for diagnosis at age 50–90 years due to the age profile of patients, and restricted to stage III and stage IV. Patients diagnosed with stage IV prostate cancer have a significantly lower estimated life expectancy than those diagnosed with stage III, across all ages. The stage-specific age distribution plot indicates a greater incidence at stage I than stages II–IV across all ages.

Figure 3 gives the stage-specific life expectancy for female patients diagnosed with cervical cancer and Hodgkin lymphoma from age 30 to 90 years. The stage-specific age distribution plot indicates a greater incidence of cervical cancer at stage I than stages II–IV among younger patients. The estimated life expectancy of patients diagnosed with stage I Hodgkin lymphoma is very similar to that of the general population, especially among younger patients. There appears to be smaller differences between patients diagnosed at different stages compared with other cancer sites.

Stage-specific life expectancy estimates are also given for female patients diagnosed with ovarian, stomach and bladder cancer at age 40–90 years. The incidence of patients diagnosed with ovarian cancer is also greater at stage I than stages II–IV among younger patients, while stages III and IV are more common among older patients. Stage II is relatively uncommon across all ages. The life expectancy estimates for patients diagnosed with stage IV stomach cancer remain very close to the grey area for all ages, indicating very poor prognosis. The stage-specific age distribution plot indicates a greater incidence at stage IV than stages I–III across all ages. The incidence of patients diagnosed with bladder cancer is greater among older patients across all stages, as indicated by the stage-specific age distribution plot.

Figure 4 gives the stage-specific life expectancy for male patients diagnosed with stomach and bladder cancer from age 40 to 90 years. The incidence of patients diagnosed with bladder cancer is greater at stage I than stages II–IV across all ages, as indicated by the stage-specific age distribution plot. There appears to be little difference in life expectancy estimates for patients diagnosed at stage II compared with those diagnosed at stage III.

Life expectancy estimates for male patients diagnosed with Hodgkin lymphoma are given for diagnosis at age 30–90 years. The estimated life expectancy of patients diagnosed with stage I Hodgkin lymphoma is very similar to that of the general population, especially among younger patients. As with female patients, there does not appear to be large differences in life expectancy between stage groups.

Online supplemental appendix E, figures A2–A11 display comparisons of 10-year relative survival estimates obtained by parametric and non-parametric methods for each of the 10 cancer sites by age group, stage and sex. The model-based estimates are very consistent with the non-parametric estimates. There is some deviation for low incident stages among young people, but otherwise there is excellent agreement.

## Discussion

This paper provides the first estimates of stage-specific life expectancy for 10 cancer sites using national English cancer registry data. Life expectancy estimates represent the whole life horizon and are well understood, largely because they are real-world metrics. Given the strength of stage at diagnosis as a prognostic factor, stage-specific estimates are a vital tool in understanding the burden of cancer. Despite substantial improvements in the recording of stage at diagnosis in cancer registries in the past 15 years in England, many cancer sites still exhibit a significant proportion of cases with missing stage information. Multiple imputation allows us to obtain stage-specific life estimates without wasting available data, while appropriately accounting for uncertainty. Stage-specific life expectancy estimates provide an overall summary metric of the impact of cancer on life expectancy, and, when coupled with a direct comparison with life expectancy estimates in the general population, provide a comprehensive picture of the overall impact of cancer on survival prospects.

Our findings showed that the estimated life expectancy for female and male patients diagnosed with stage IV colorectal cancer is vastly lower than for stages I–III. Online supplemental appendix G, table A5 indicates that the predicted age at death for female patients diagnosed with stage IV colorectal cancer at age 40 is 46.3 years. However, the predicted age at death for females diagnosed with stage I–III colorectal cancer at age 40 is 71.3 years or greater. Other research has also found a significant prognosis reduction for patients with stage IV versus stages I–III,^29 39^ with the pattern likely being driven by the common metastatic sites.^40^ This finding highlights the potential benefits of earlier diagnosis in terms of achieving stark improvements in life expectancy for patients with colorectal cancer. The rate of early-onset colorectal cancer (younger than 50 years of age at diagnosis) has been increasing in recent years, and research suggests that England experienced the fourth fastest rise in the rate of early-onset colorectal cancer (3.6% per year) in the 10-year period to 2017.^41 42^ Research also suggests many of these patients are diagnosed at a more advanced stage.^43^

Similar results can be seen for bladder cancer. Our findings also highlight the greater life expectancy for female patients diagnosed with stage I cervical and stomach cancer compared with stages II–IV.

Although age, stage and sex are important prognostic factors, there are other factors such as deprivation, comorbidity, treatment and histological information that are not included in the models, but affect cancer survival. The aim of this paper was to provide up-to-date estimates that reflect the survival of patients, rather than a hypothetical cohort that is used for making fair comparisons. As such, if deprivation varies greatly between two stage groups, it is possible that there will be variation between the stage-specific estimates even if the cancer-specific survival is very similar. The estimates reflect a group of patients of the same sex, stage and age at diagnosis, and therefore an individual’s prognosis may look different depending on their deprivation quintile, comorbidity status and histological data. Other factors such as treatment options and healthcare system may also impact life expectancy, and hence although the overall patterns may be generalisable to other populations, these results are specific to patients recorded in the English cancer registry during the study period. Some sites, such as lung cancer, exhibit a small dip in life expectancy among younger patients. It is possible that this shape is caused by the variation in lung cancer subtype by age at diagnosis. Subtype distribution may be different for early-onset patients, and also varies by smoking status. Future work could include the consideration of further variables such as comorbidity status and smoking status in both the imputation model and analysis models. We did not include any postdiagnosis information such as histological or treatment data in this analysis. However, age and stage are key predictors of treatment. Further future work could retrospectively validate the presented methodology using more complete regional data.

This study is the first of its kind to use national English data. There are a number of strengths to this study. Use of the national data provides a large population size, with a high level of completeness. As displayed in online supplemental appendix E, there is very good agreement between the model-based estimates and non-parametric estimates. Figures1^4^ detailing life expectancy differ from typical life expectancy plots in the choice of y-axis. It is common for remaining life years to be shown on the y-axis, rather than attained age at death as displayed here. Presenting attained age at death may further enhance the interpretability of the life expectancy metric. The proportion of life lost can also be visually approximated. Even if a patient diagnosed at 30 years of age has more estimated remaining life years than a patient diagnosed at 70 years of age, their expected age at death may be younger. Given life expectancy for patients with cancer is a function of both other-cause and cancer-specific mortality, differences in life expectancy may be driven by non-cancer-related differences. Comparisons by sex illustrate this since women are typically expected to live longer than men in the general population. Life expectancy is conditional on age and hence may increase as age at diagnosis increases, even if there is no real difference in cancer-specific survival between each unit increase in age. By including the stage-specific age distribution plot alongside the life expectancy figures, ages where cases are sparse can be quickly identified, providing further understanding of the estimates. The age profile can vary greatly between cancer sites. For example, among female patients diagnosed in 2017–2019, the median age of diagnosis for cervical cancer is 44.5, whereas the median age of diagnosis for female patients with bladder cancer is 77.5. Given age is a prognostic factor and also associated with conditional life expectancy in the general population, the age profile of cases can be very impactful on the shape and stability of the estimated life expectancy curves.

A potential limitation of this study is that it is not possible to achieve compatibility between the imputation model and analysis model since the analysis models are stratified by stage at diagnosis. This is a trade-off to improve model flexibility and therefore model fit without having to make strong assumptions regarding the effect of stage. The selected imputation model is based on the most complex candidate analysis model, and hence in cases where a simpler analysis model is fit there will be further discrepancy between the imputation model and analysis model. There may be covariates or relationships included in the imputation model but not in the analysis model. The stage value assigned to a particular patient with missing stage at diagnosis information may vary between imputed datasets, by definition. Since separate analysis models are fit for each stage group, it is likely that the analysis models are fit to slightly varying sample sizes across the imputations. Great effort was made to ensure that the same model is fit to each imputed dataset where possible, and this included fixing the location of the knots. However, in the case where a simpler model must be fit to a particular imputed dataset, the simpler model may have fewer df and therefore different knot locations.

Multiple imputation requires the assumption that missing data are missing at random (MAR), that is, the probability of stage at diagnosis being missing depends only on other observed variables and not on the true missing value of stage at diagnosis. Missing stage is often associated with older patients and higher mortality, which are captured in the imputation model by the age term, Nelson-Aalen estimator and the event indicator. Survival variables are key for imputing time-to-event data. Trends in stage completeness are associated with calendar year, which is also included in the model. The MAR assumption is untestable; hence, we acknowledge that the inclusion of further information, such as comorbidity, histological and treatment data, may improve the performance of the imputation model and improve the validity of the assumption. However, previous research indicates that stage-specific long-term relative survival estimates are robust to poor imputation of stage at diagnosis provided sufficiently high completeness of stage information within the period window.^14^ In these data, the completeness of stage information is higher in the period window and recent years. The short-term survival experiences are highly impactful on the overall survival curve. The patients with less complete stage information are typically diagnosed many years ago, and are contributing to the long-term estimates rather than the short-term estimates.

The imputation model uses the all-cause event indicator and therefore may not fully account for the competing risks nature of the analysis. Although other-cause mortality is not directly accounted for, factors that affect other-cause mortality (sex, age and year) are included in the imputation model. Other research considering the imputation of stage for estimating excess HRs recommends including the Nelson-Aalen cumulative hazard estimate and the event indicator,^44^ as we have in this study.

Stage-specific life expectancy provides an interpretable metric, which can be directly compared with general population life expectancy estimates to understand the impact of cancer. We analyse a range of cancer sites using national data for England, using appropriate methodology to handle historical incompleteness of stage information. These results represent the estimated stage-specific life expectancy of patients recently diagnosed in England, but the statistical methodology could be extended to other populations. This work highlights target groups for reducing mortality and facilitates the continued monitoring of trends in cancer burden over time. Comparisons across stage-specific estimates for a given age at diagnosis can motivate efforts to attain life expectancy gains through early diagnosis initiatives.

In conclusion, we have highlighted stark differences in stage-specific life expectancy for a range of cancer sites in England using national data for the first time. Clinically, these differences in prognosis are largely driven by the treatment options available, which depend on cancer stage and other patient characteristics at diagnosis. Our work highlights that policy efforts to decrease late-stage cancer diagnosis will lead to marked gains in life expectancy. The same analytical approaches can be used to evaluate patient group inequalities, such as by socioeconomic group or across geographies, to quantify the population and individual benefit of earlier cancer diagnosis.

## Supplementary material

10.1136/bmjonc-2025-000999 online supplemental file 1

## Data Availability

Data may be obtained from a third party and are not publicly available.
