# Supplementary material for: Estimating the impact of cancer diagnosis on life expectancy by stage at diagnosis: population-based estimates for a range of cancer sites in England
Source: BMJ Oncol. 2026 Apr 30;5(1):e000999. doi: 10.1136/bmjonc-2025-000999 (PMC13140960; doi:10.1136/bmjonc-2025-000999)
Supplement: online supplemental file 1 [file bmjonc-5-1-s001.docx]

# APPENDICES

## Appendix A

|  | Year of Follow-Up | | | | | | | | | | | | | | |
| --- | --- | --- | --- | --- | --- | --- | --- | --- | --- | --- | --- | --- | --- | --- | --- |
| Year of Diagnosis |  | 2007 | 2008 | 2009 | 2010 | 2011 | 2012 | 2013 | 2014 | 2015 | 2016 | **2017** | **2018** | **2019** | **2020** |
|  | 2007 | 1 | 1-2 | 2-3 | 3-4 | 4-5 | 5-6 | 6-7 | 7-8 | 8-9 | 9-10 | 10 |  |  |  |
|  | 2008 |  | 1 | 1-2 | 2-3 | 3-4 | 4-5 | 5-6 | 6-7 | 7-8 | 8-9 | 9-10 | 10 |  |  |
|  | 2009 |  |  | 1 | 1-2 | 2-3 | 3-4 | 4-5 | 5-6 | 6-7 | 7-8 | 8-9 | 9-10 | 10 |  |
|  | 2010 |  |  |  | 1 | 1-2 | 2-3 | 3-4 | 4-5 | 5-6 | 6-7 | 7-8 | 8-9 | 9-10 | 10 |
|  | 2011 |  |  |  |  | 1 | 1-2 | 2-3 | 3-4 | 4-5 | 5-6 | 6-7 | 7-8 | 8-9 | 9-10 |
|  | 2012 |  |  |  |  |  | 1 | 1-2 | 2-3 | 3-4 | 4-5 | 5-6 | 6-7 | 7-8 | 8-9 |
|  | 2013 |  |  |  |  |  |  | 1 | 1-2 | 2-3 | 3-4 | 4-5 | 5-6 | 6-7 | 7-8 |
|  | 2014 |  |  |  |  |  |  |  | 1 | 1-2 | 2-3 | 3-4 | 4-5 | 5-6 | 6-7 |
|  | 2015 |  |  |  |  |  |  |  |  | 1 | 1-2 | 2-3 | 3-4 | 4-5 | 5-6 |
|  | 2016 |  |  |  |  |  |  |  |  |  | 1 | 1-2 | 2-3 | 3-4 | 4-5 |
|  | 2017 |  |  |  |  |  |  |  |  |  |  | 1 | 1-2 | 2-3 | 3-4 |
|  | 2018 |  |  |  |  |  |  |  |  |  |  |  | 1 | 1-2 | 2-3 |
|  | 2019 |  |  |  |  |  |  |  |  |  |  |  |  | 1 | 1-2 |
|  | 2020 |  |  |  |  |  |  |  |  |  |  |  |  |  | 1 |

Figure A 1: Period analysis schematic with period window spanning January 1st 2017 - February 29th 2020. All follow-up ceased after 10 years.

## Appendix B

Table A 1: Distribution of staging system used for cases diagnosed 2017-2019. Values are percentages, given by cancer site, and excluding unstaged cases.

| Staging System | Cancer Site | | | | | | | | | |
| --- | --- | --- | --- | --- | --- | --- | --- | --- | --- | --- |
|  | Bladder | Breast | Cervical | Colorectal | Hodgkin  Lymphoma | Lung | Melanoma | Prostate | Ovarian | Stomach |
| Unknown | 1 | 1 | - | 1 | 1 | 1 | 1 | 1 | <1 | 1 |
| AJCC 7 | - | <1 | - | - | - | <1 | 30 | <1 | - | - |
| AJCC 8 | - | <1 | - | - | - | <1 | <1 | - | - | - |
| Ann Arbor | - | - | - | - | 99 | - | - | - | - | - |
| ENETS 2007 | - | - | - | 1 | - | - | - | - | - | 2 |
| FIGO | - | - | 100 | - | - | - | - | - | 84 | - |
| UICC 5 | <1 | <1 | - | 31 | - | <1 | <1 | <1 | - | <1 |
| UICC 6 | <1 | <1 | - | < 1 | - | <1 | <1 | <1 | <1 | <1 |
| UICC 7 | 35 | 33 | - | 1 | <1 | 33 | 1 | 30 | 5 | 33 |
| UICC 8 | 64 | 66 | - | 66 | <1 | 66 | 67 | 69 | 11 | 63 |

## Appendix C

Missing cancer stage at diagnosis was imputed under a multinomial logistic model with 50 impute datasets. Stage at diagnosis was only imputed for individuals who contribute person-time to the period window. The variables selected for the imputation model are: sex, age, year of diagnosis, deprivation, an interaction term between sex and age, and interactions terms between calendar year and age, sex and deprivation. Further variables include the event indicator, the Nelson–Aalen estimate of cumulative hazard (H) and H1, which is related to H. The imputed model also includes interaction terms between the event indicator and survival time, age and H, age and H1, sex and H and sex and H1 as recommended by the time-varying effects approximate approach of Keogh and Morris (9). Keogh and Morris define H1 similar to the Nelson–Aalen cumulative hazard estimator as $H_{1}\left( t \right)=\sum_{t\leq T} t\frac{d(t)}{n(t)}$ where *d(t*) and *n(t)* are the number of deaths and number at risk at time *t*. The event indicator is 0 if alive at the end of follow-up and 1 if dead due to any cause.

Estimating life expectancy requires the all-cause survival curve to reach 0, but this is rarely observed in observational cohort data. Since the excess hazard often tends to 0 or a low value in the long-term, the relative survival framework enables stable extrapolation. In the net survival framework, the overall mortality rate is expressed as the sum of the expected hazard (in a representative population that is free of cancer) and the excess hazard due to the cancer under study. The expected hazard is assumed to be known from population lifetables, leaving the excess hazard to be estimated. We can estimate life expectancy from these excess hazard models and hence reliably estimate life expectancy.

The baseline cumulative excess hazard was modelled using natural splines with 5 degrees of freedom. Flexible parametric models allow complex baseline cumulative excess hazard functions to be captured, without making strong distributional assumptions. The selected flexible parametric models included age and sex as main covariates, an interaction between age and sex, and time dependent effects of age and sex. Age was modelled using natural splines with 4 degrees of freedom. Age was winsorised at the 1st and 99th percentiles to provide more stability in the tails of the data. Data is often sparse in the tails and so the effect due to age is constrained to be constant in the upper and lower 1 percentiles of the data (11). Time-dependent effects of age were captured using natural splines with 2 degrees of freedom. New spline terms were generated for age with only 2 degrees of freedom, instead of the 4 degrees of freedom used for the main effects. This specification concerns the preferred model. Other, simpler models were also used where necessary.

Since stage at diagnosis is an imputed variable and the flexible parametric models are stratified by stage at diagnosis, the number of observations included in each model will naturally vary between imputations. Under our approach, the post-estimation predictions are pooled rather than the model coefficients. Due to the changing number of observations and in turn potential change in covariate and event distribution, the knot locations for the baseline hazard, effect of time, effect of age and time-dependent effect of age are fixed across the imputation datasets. These locations were chosen separately by stage by fitting an excess hazard model to the complete case data. This also applies to the winsor points for the age variable.

## Appendix D

Table A 2: Candidate models for bladder cancer, colorectal cancer, Hodgkin Lymphoma, lung cancer, melanoma and stomach cancer. All models include age and sex as main effects. * Degrees of freedom (DF) splines modelling baseline. ^†^ Degrees of freedom for modelling main effects of age. W^~^ Symmetrical winsor points for age (%). ^‡^ Inclusion of the age*sex interaction term. The degrees of freedom for splines modelling age for time-dependent effects (TDE) and interaction terms are always 2 if present. The degrees of freedom for splines modelling time for time-dependent effects are always 4 if present. The frequency of fitted models is given for each stage and cancer site for the 50 imputed datasets.

| Model | | | | Cancer Site | | | | | | | | | | | | | | | | | | | | | | | |
| --- | --- | --- | --- | --- | --- | --- | --- | --- | --- | --- | --- | --- | --- | --- | --- | --- | --- | --- | --- | --- | --- | --- | --- | --- | --- | --- | --- |
| DF  (Base*, Age^†^) | W^~^ | Int^‡^ | TDE | Bladder | | | | Colorectal | | | | Hodgkin  Lymphoma | | | | Lung | | | | Melanoma | | | | Stomach | | | |
|  |  |  |  | I | II | III | IV | I | II | III | IV | I | II | III | IV | I | II | III | IV | I | II | III | IV | I | II | III | IV |
| (5, 4) | 1 | Yes | Age, Sex | 50 | 50 | 14 | 40 | 49 | 50 | 50 | 50 | 0 | 0 | 50 | 50 | 50 | 50 | 50 | 50 | 0 | 50 | 50 | 0 | 49 | 50 | 49 | 46 |
| (5, 4) | 1 | Yes | Age | 0 | 0 | 17 | 7 | 0 | 0 | 0 | 0 | 0 | 0 | 0 | 0 | 0 | 0 | 0 | 0 | 0 | 0 | 0 | 0 | 0 | 0 | 1 | 1 |
| (5, 4) | 2 | Yes | Age | 0 | 0 | 14 | 3 | 1 | 0 | 0 | 0 | 0 | 0 | 0 | 0 | 0 | 0 | 0 | 0 | 0 | 0 | 0 | 0 | 1 | 0 | 0 | 3 |
| (5, 4) | 2 | No | Age | 0 | 0 | 0 | 0 | 0 | 0 | 0 | 0 | 0 | 0 | 0 | 0 | 0 | 0 | 0 | 0 | 0 | 0 | 0 | 0 | 0 | 0 | 0 | 0 |
| (5, 4) | 2 | Yes | - | 0 | 0 | 5 | 0 | 0 | 0 | 0 | 0 | 0 | 50 | 0 | 0 | 0 | 0 | 0 | 0 | 0 | 0 | 0 | 50 | 0 | 0 | 0 | 0 |
| (5, 4) | 2 | No | - | 0 | 0 | 0 | 0 | 0 | 0 | 0 | 0 | 0 | 0 | 0 | 0 | 0 | 0 | 0 | 0 | 0 | 0 | 0 | 0 | 0 | 0 | 0 | 0 |
| (4, 4) | 2 | No | - | 0 | 0 | 0 | 0 | 0 | 0 | 0 | 0 | 0 | 0 | 0 | 0 | 0 | 0 | 0 | 0 | 50 | 0 | 0 | 0 | 0 | 0 | 0 | 0 |
| (4, 3) | 2 | No | - | 0 | 0 | 0 | 0 | 0 | 0 | 0 | 0 | 38 | 0 | 0 | 0 | 0 | 0 | 0 | 0 | 0 | 0 | 0 | 0 | 0 | 0 | 0 | 0 |
| (3, 3) | 2 | No | - | 0 | 0 | 0 | 0 | 0 | 0 | 0 | 0 | 12 | 0 | 0 | 0 | 0 | 0 | 0 | 0 | 0 | 0 | 0 | 0 | 0 | 0 | 0 | 0 |
| (2, 3) | 2 | No | - | 0 | 0 | 0 | 0 | 0 | 0 | 0 | 0 | 0 | 0 | 0 | 0 | 0 | 0 | 0 | 0 | 0 | 0 | 0 | 0 | 0 | 0 | 0 | 0 |

Table A 3: Candidate models for breast cancer, cervical cancer, ovarian cancer and prostate cancer. All models include age as main effect. * Degrees of freedom (DF) splines modelling baseline. ^†^ Degrees of freedom for modelling main effects of age. W^~^ Symmetrical winsor points for age (%). The degrees of freedom for splines modelling age for time-dependent effects (TDE) are always 2 if present. The degrees of freedom for splines modelling time for time-dependent effects are always 4 if present. The frequency of fitted models is given for each stage and cancer site for the 50 imputed datasets.

| Model | | | Cancer Site | | | | | | | | | | | | | | | |
| --- | --- | --- | --- | --- | --- | --- | --- | --- | --- | --- | --- | --- | --- | --- | --- | --- | --- | --- |
| DF  (Base*, Age^†^) | W^~^ | TDE | Breast | | | | Cervical | | | | Prostate | | | | Ovarian | | | |
|  |  |  | I | II | III | IV | I | II | III | IV | I | II | III | IV | I | II | III | IV |
| (5, 4) | 1 | Age | 50 | 50 | 50 | 50 | 49 | 35 | 0 | 0 |  | | 43 | 50 | 50 | 0 | 0 | 50 |
| (5, 4) | 2 | Age | 0 | 0 | 0 | 0 | 0 | 0 | 0 | 0 |  |  | 3 | 0 | 0 | 0 | 50 | 0 |
| (5, 4) | 2 | - | 0 | 0 | 0 | 0 | 1 | 15 | 50 | 50 |  |  | 4 | 0 | 0 | 50 | 0 | 0 |
| (4, 4) | 2 | - | 0 | 0 | 0 | 0 | 0 | 0 | 0 | 0 |  |  | 0 | 0 | 0 | 0 | 0 | 0 |
| (4, 3) | 2 | - | 0 | 0 | 0 | 0 | 0 | 0 | 0 | 0 |  |  | 0 | 0 | 0 | 0 | 0 | 0 |
| (3, 3) | 2 | - | 0 | 0 | 0 | 0 | 0 | 0 | 0 | 0 |  |  | 0 | 0 | 0 | 0 | 0 | 0 |

## Appendix E


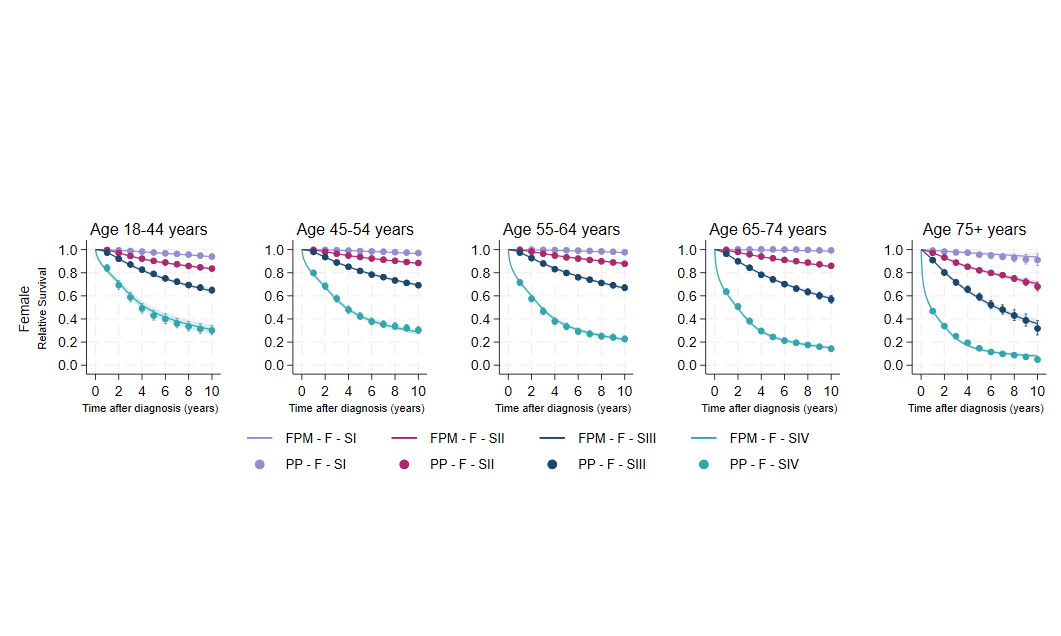


Figure A 2: Comparison of relative survival estimates obtained from flexible parametric models (FPM) and the non-parametric Pohar-Perme estimator (PP) by age group and stage for female patients diagnosed with breast cancer with 95% confidence intervals. SI-SIV denotes stage I to stage IV respectively.


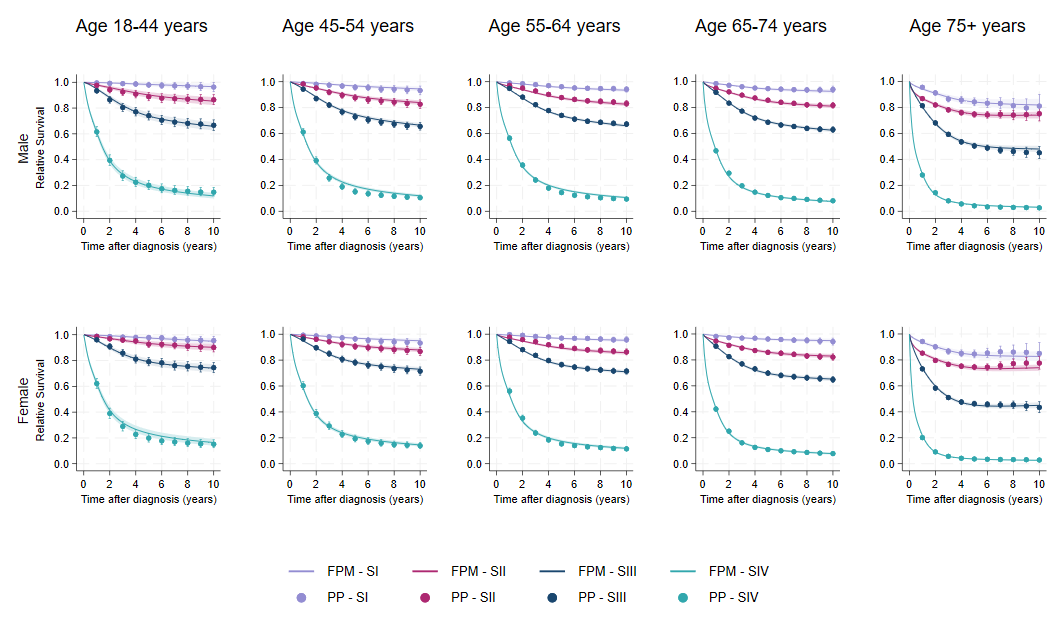


Figure A 3: Comparison of relative survival estimates obtained from flexible parametric models (FPM) and the non-parametric Pohar-Perme estimator (PP) by age group, stage and sex for patients diagnosed with colorectal cancer with 95% confidence intervals. SI-SIV denotes stage I to stage IV respectively.


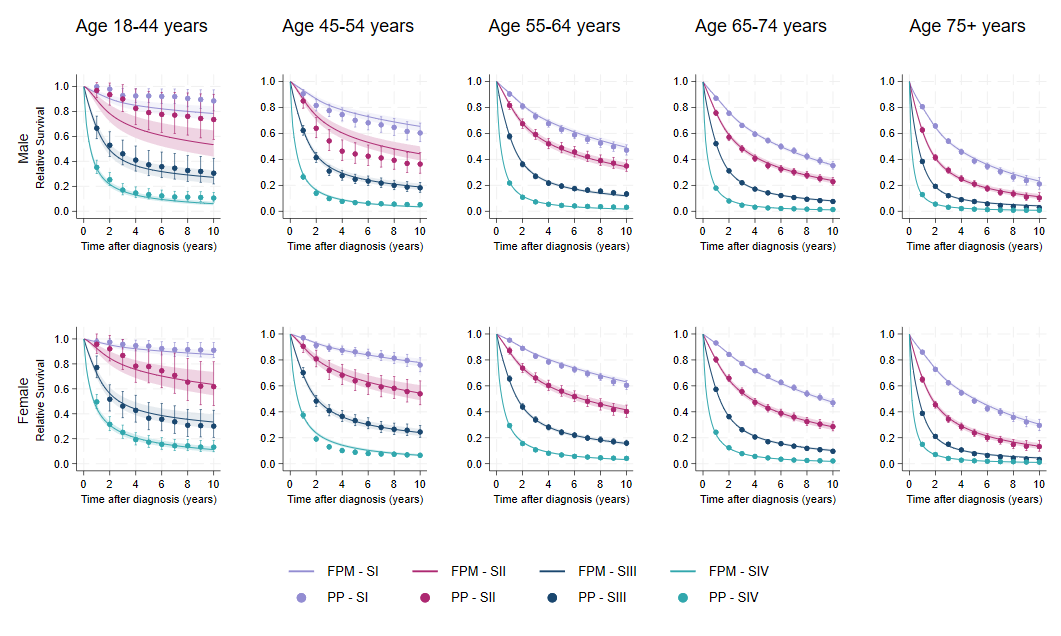


Figure A 4: Comparison of relative survival estimates obtained from flexible parametric models (FPM) and the non-parametric Pohar-Perme estimator (PP) by age group, stage and sex for patients diagnosed with lung cancer with 95% confidence intervals. SI-SIV denotes stage I to stage IV respectively.


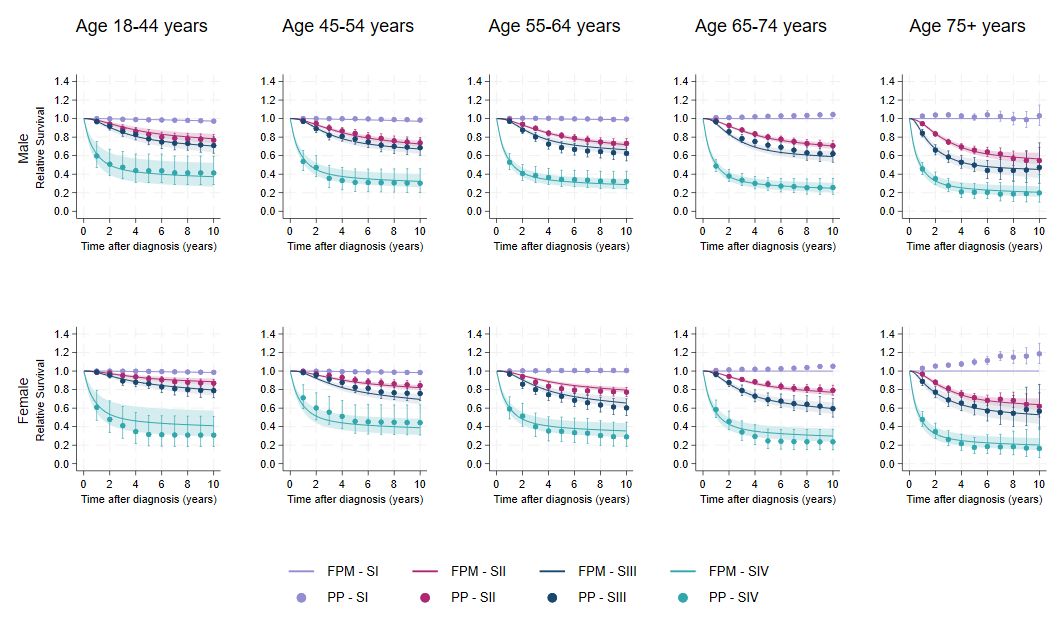


Figure A 5: Comparison of relative survival estimates obtained from flexible parametric models (FPM) and the non-parametric Pohar-Perme estimator (PP) by age group, stage and sex for patients diagnosed with melanoma with 95% confidence intervals. SI-SIV denotes stage I to stage IV respectively.


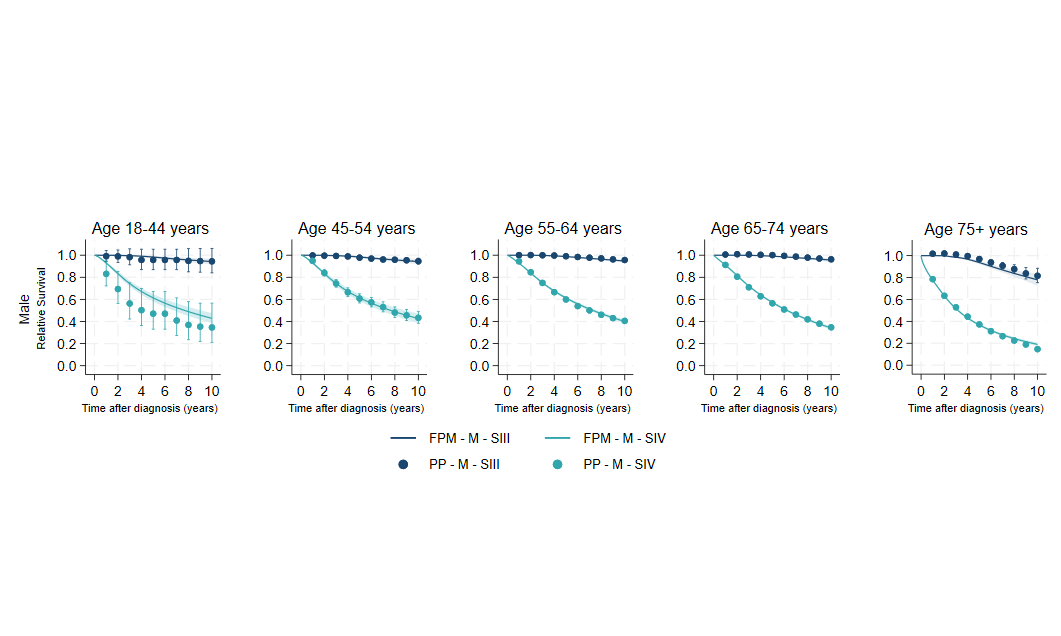


Figure A 6: Comparison of relative survival estimates obtained from flexible parametric models (FPM) and the non-parametric Pohar-Perme estimator (PP) by age group and stage for male patients diagnosed with prostate cancer with 95% confidence intervals. SIII and SIV denotes stage III and stage IV respectively.


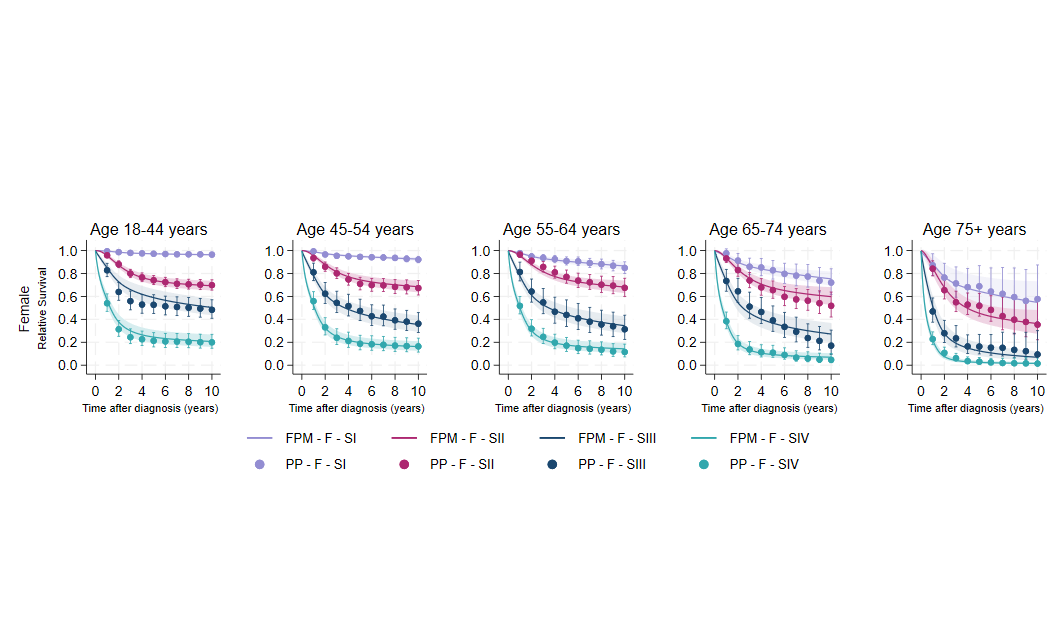


Figure A 7: Comparison of relative survival estimates obtained from flexible parametric models (FPM) and the non-parametric Pohar-Perme estimator (PP) by age group and stage for female patients diagnosed with cervical cancer with 95% confidence intervals. SI-SIV denotes stage I to stage IV respectively.


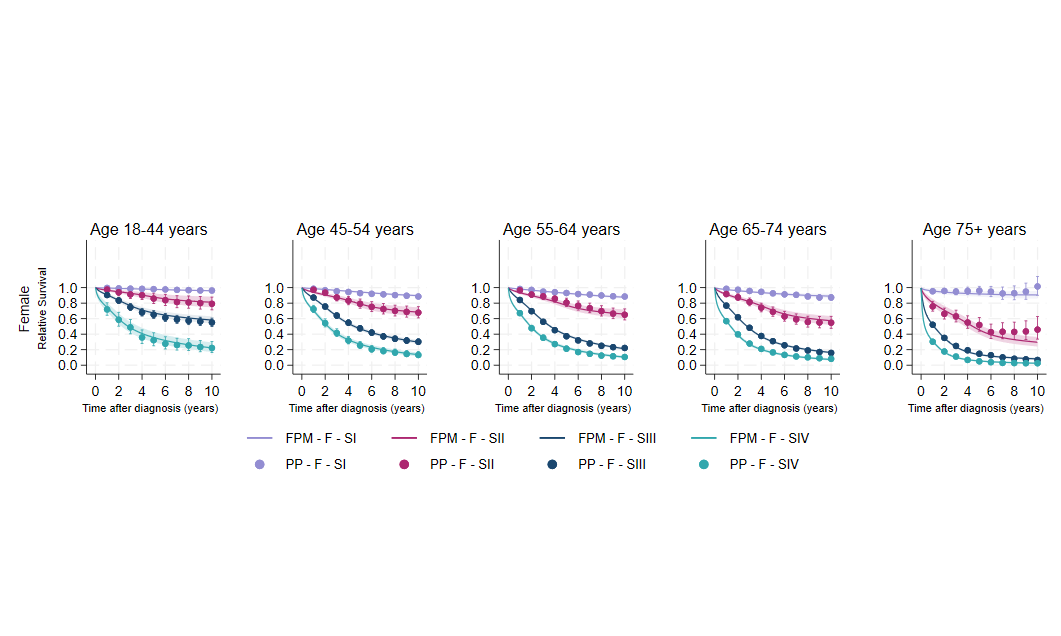


Figure A 8: Comparison of relative survival estimates obtained from flexible parametric models (FPM) and the non-parametric Pohar-Perme estimator (PP) by age group and stage for female patients diagnosed with ovarian cancer with 95% confidence intervals. SI-SIV denotes stage I to stage IV respectively.


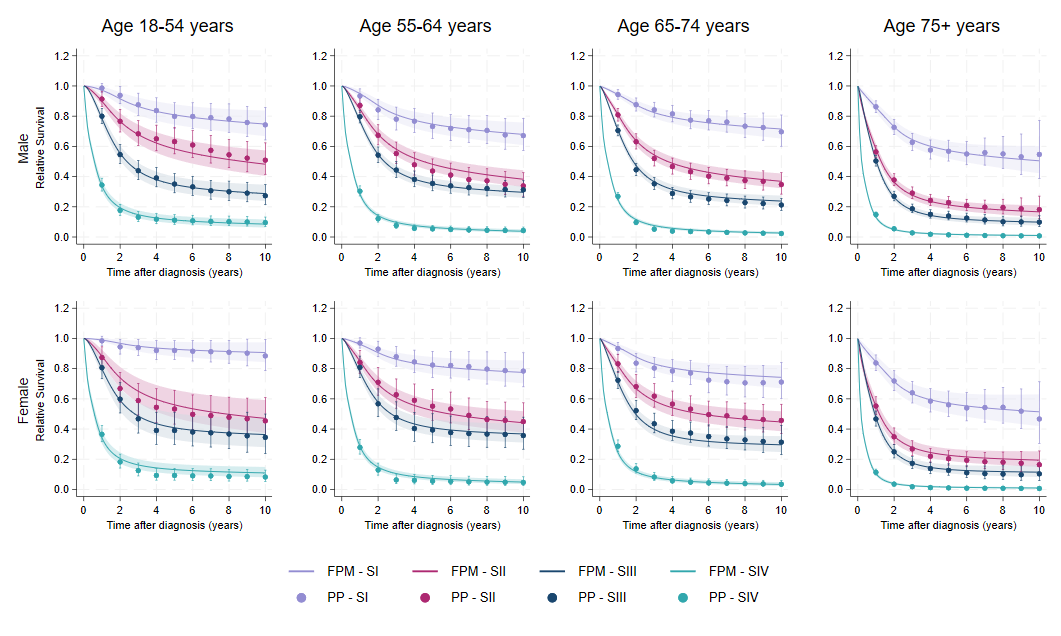


Figure A 9: Comparison of relative survival estimates obtained from flexible parametric models (FPM) and the non-parametric Pohar-Perme estimator (PP) by age group, stage and sex for patients diagnosed with stomach cancer with 95% confidence intervals. SI-SIV denotes stage I to stage IV respectively.


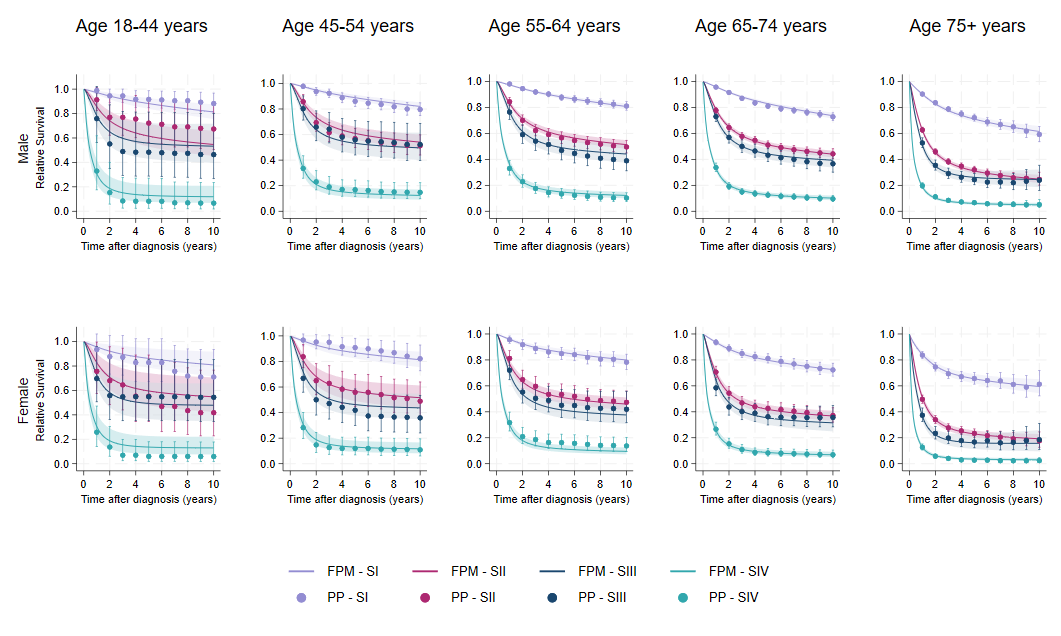


Figure A 10: Comparison of relative survival estimates obtained from flexible parametric models (FPM) and the non-parametric Pohar-Perme estimator (PP) by age group, stage and sex for patients diagnosed with bladder cancer with 95% confidence intervals. SI-SIV denotes stage I to stage IV respectively.


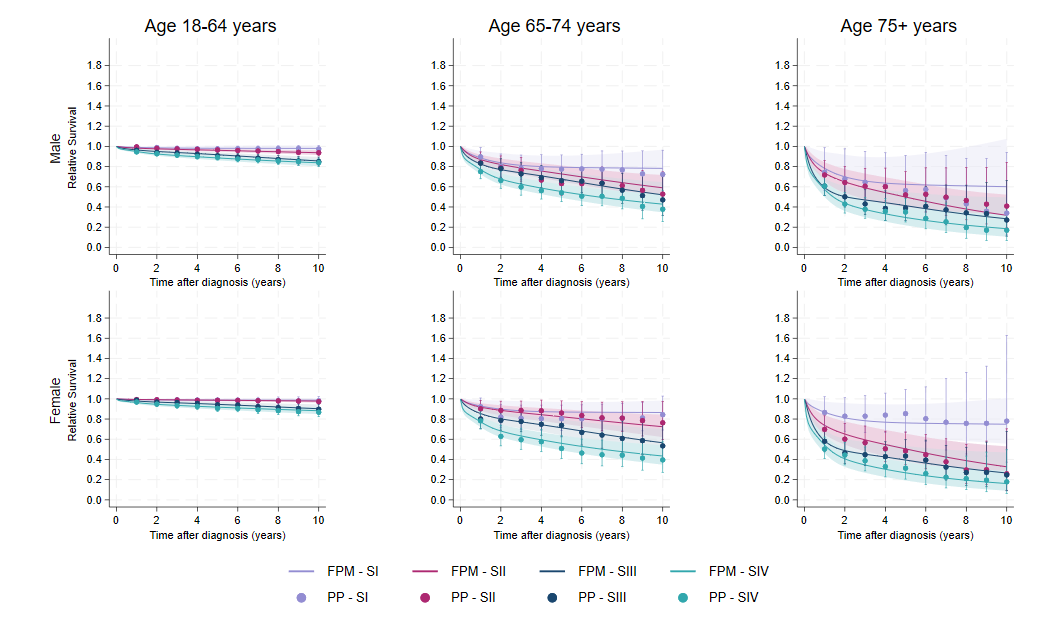


Figure A 11: Comparison of relative survival estimates obtained from flexible parametric models (FPM) and the non-parametric Pohar-Perme estimator (PP) by age group, stage and sex for patients diagnosed with Hodgkin Lymphoma with 95% confidence intervals. SI-SIV denotes stage I to stage IV respectively.

## Appendix F

Table A 4: Baseline characteristics for patients diagnosed in 2017 to 2019. Demographics marked with * are given as median (25^th^ percentile, 75^th^ percentile) and those marked with † are given as N (%). ‡ Deprivation graded from 1 (most deprived) to 5 (least deprived). ** Percentages for stages I-IV sum to 100, with separate percent given for unknown stage.

|  | | Cancer Site | | | | | | | | | |
| --- | --- | --- | --- | --- | --- | --- | --- | --- | --- | --- | --- |
|  |  | Bladder | Breast | Cervical | Colorectal | Hodgkin Lymphoma | Lung | Melanoma | Ovarian | Prostate | Stomach |
| Total (*N*) | | 26,010 | 129,226 | 7,940 | 104,391 | 4,645 | 115,037 | 40,995 | 18,529 | 139,249 | 15,756 |
| Age (*years*) * | | 76 (69, 83) | 63 (52, 73) | 44 (34, 59) | 72 (63, 81) | 47 (29, 66) | 73 (66, 80) | 66 (52, 76) | 66 (54, 75) | 71 (64, 77) | 74 (65, 82) |
| Sex † | Male | 19,058 (73) | 0 (0) | 0 (0) | 58,188 (56) | 2,700 (58) | 60,073 (52) | 20,812 (51) | 0 (0) | 139,249 (100) | 10,272 (65) |
|  | Female | 6,952 (27) | 129,226 (100) | 7,940 (100) | 46,203 (44) | 1,945 (42) | 54,964 (48) | 20,183 (49) | 18,529 (100) | 0 (0) | 5,484 (35) |
| Stage †** | I | 9,863 (47) | 50,766 (44) | 4,056 (61) | 16,964 (19) | 514 (13) | 21,898 (21) | 25,563 (68) | 5,346 (35) | 45,218 (38) | 1,464 (12) |
|  | II | 5,925 (28) | 48,664 (42) | 1,350 (20) | 23,229 (25) | 1,357 (33) | 8,490 (8) | 7,823 (21) | 1,026 (7) | 19,277 (16) | 1,812 (15) |
|  | III | 2,390 (11) | 10,903 (9) | 526 (8) | 28,145 (31) | 942 (23) | 22,514 (21) | 2,866 (8) | 5,454 (36) | 31,040 (26) | 2,759 (23) |
|  | IV | 2,907 (14) | 5,986 (5) | 689 (10) | 23,199 (25) | 1,272 (31) | 52,943 (50) | 1,101 (3) | 3,539 (23) | 24,609 (20) | 5,902 (49) |
|  | Unknown | 4,925 (19) | 12,907 (10) | 1,319 (17) | 12,854 (12) | 560 (12) | 9,192 (8) | 3,642 (9) | 3,164 (17) | 19,105 (14) | 3,819 (24) |
| Deprivation † ‡ | 1 (Most) | 4,520 (17) | 20,069 (16) | 2,150 (27) | 17,084 (16) | 930 (20) | 30,209 (26) | 4,448 (11) | 3,281 (18) | 18,216 (13) | 3,430 (22) |
|  | 2 | 4,937 (19) | 23,358 (18) | 1,732 (22) | 18,999 (18) | 954 (21) | 24,506 (21) | 6,365 (16) | 3,471 (19) | 23,971 (17) | 3,255 (21) |
|  | 3 | 5,420 (21) | 27,040 (21) | 1,561 (20) | 21,898 (21) | 988 (21) | 22,506 (20) | 8,549 (21) | 3,888 (21) | 29,568 (21) | 3,161 (20) |
|  | 4 | 5,737 (22) | 28,921 (22) | 1,320 (17) | 23,095 (22) | 914 (20) | 20,585 (18) | 10,145 (25) | 3,920 (21) | 32,788 (24) | 3,066 (19) |
|  | 5 (Least) | 5,396 (21) | 29,838 (23) | 1,177 (15) | 23,315 (22) | 859 (18) | 17,231 (15) | 11,488 (28) | 3,969 (21) | 34,706 (25) | 2,844 (18) |

## Appendix G

Table A 5: Stage-specific predicted age at death (95% CI) for female patients by cancer site for a range of ages at diagnosis.

| Age | Stage | General  Population | Bladder | Breast | Cervical | Colorectal | Hodgkin  Lymphoma | Lung | Melanoma | Ovarian | Stomach |
| --- | --- | --- | --- | --- | --- | --- | --- | --- | --- | --- | --- |
| 40 | I | 84.2 | 72.8 | 76.0 | 81.4 | 79.5 | 84.0 | 76.8 | 80.8 | 80.0 | 81.5 |
|  |  |  | (67.1, 79.7) | (73.5, 78.7) | (80.3, 82.5) | (75.8, 83.6) | (83.3, 84.7) | (74.3, 79.4) | (78.8, 83.0) | (78.2, 81.9) | (77.3, 86.1) |
|  | II |  | 63.1 | 72.6 | 68.8 | 78.3 | 83.3 | 64.4 | 76.2 | 71.9 | 59.3 |
|  |  |  | (57.3, 70.8) | (71.5, 73.8) | (65.8, 72.3) | (76.5, 80.1) | (82.0, 84.6) | (59.7, 70.2) | (73.8, 78.7) | (68.1, 76.2) | (52.7, 69.4) |
|  | III |  | 61.6 | 61.7 | 57.3 | 71.3 | 76.7 | 53.3 | 70.3 | 58.6 | 54.9 |
|  |  |  | (55.6, 69.9) | (59.9, 63.6) | (53.6, 62.2) | (69.7, 73.0) | (71.0, 83.4) | (50.9, 56.3) | (65.8, 75.6) | (56.4, 61.2) | (49.5, 63.3) |
|  | IV |  | 46.2 | 50.2 | 47.9 | 46.3 | 78.2 | 44.2 | 57.2 | 46.8 | 46.5 |
|  |  |  | (43.7, 50.4) | (48.8, 51.7) | (45.9, 50.5) | (45.5, 47.3) | (75.2, 81.5) | (43.5, 45.1) | (52.6, 63.4) | (45.4, 48.6) | (44.4, 49.5) |
| 50 | I | 84.7 | 76.7 | 81.1 | 81.2 | 82.1 | 84.4 | 75.5 | 82.8 | 79.5 | 79.9 |
|  |  |  | (73.9, 79.8) | (80.1, 82.1) | (79.9, 82.5) | (80.9, 83.4) | (83.4, 85.3) | (73.9, 77.2) | (81.8, 83.9) | (78.1, 81.0) | (76.5, 83.6) |
|  | II |  | 67.8 | 78.2 | 73.1 | 79.8 | 83.3 | 67.1 | 77.1 | 72.4 | 64.8 |
|  |  |  | (64.7, 71.4) | (77.7, 78.7) | (70.6, 75.8) | (79.0, 80.6) | (81.6, 85.0) | (64.8, 69.6) | (75.5, 78.7) | (69.7, 75.4) | (61.7, 68.8) |
|  | III |  | 65.7 | 70.4 | 61.2 | 74.7 | 76.8 | 58.3 | 72.2 | 59.1 | 63.4 |
|  |  |  | (62.4, 70.0) | (69.5, 71.4) | (58.8, 64.1) | (74.0, 75.5) | (72.2, 82.4) | (57.3, 59.4) | (69.5, 75.3) | (58.3, 60.0) | (60.6, 66.9) |
|  | IV |  | 54.5 | 58.9 | 55.9 | 55.2 | 77.4 | 52.6 | 63.4 | 54.8 | 53.1 |
|  |  |  | (53.2, 56.3) | (58.1, 59.7) | (54.6, 57.6) | (54.8, 55.6) | (74.2, 80.9) | (52.4, 52.9) | (60.6, 66.9) | (54.2, 55.4) | (52.4, 54.0) |
| 60 | I | 85.6 | 80.1 | 84.1 | 81.7 | 84.3 | 84.6 | 75.3 | 85.2 | 81.9 | 79.8 |
|  |  |  | (79.0, 81.3) | (83.6, 84.6) | (80.4, 83.0) | (83.8, 84.8) | (83.3, 86.1) | (74.6, 76.0) | (84.8, 85.6) | (81.1, 82.8) | (77.7, 82.2) |
|  | II |  | 72.3 | 81.6 | 77.6 | 81.9 | 82.6 | 70.7 | 80.0 | 76.9 | 71.6 |
|  |  |  | (71.1, 73.7) | (81.2, 81.9) | (75.9, 79.4) | (81.4, 82.4) | (80.4, 85.1) | (69.9, 71.6) | (79.0, 81.0) | (75.2, 78.8) | (69.9, 73.6) |
|  | III |  | 70.2 | 75.8 | 69.1 | 78.4 | 78.0 | 64.8 | 76.6 | 66.2 | 70.3 |
|  |  |  | (68.4, 72.4) | (75.1, 76.6) | (67.5, 71.2) | (78.0, 78.8) | (74.8, 81.8) | (64.5, 65.1) | (74.8, 78.5) | (65.8, 66.6) | (68.8, 72.1) |
|  | IV |  | 63.0 | 66.3 | 64.4 | 63.8 | 76.6 | 61.5 | 69.5 | 63.7 | 61.8 |
|  |  |  | (62.3, 63.8) | (65.9, 66.7) | (63.4, 65.7) | (63.6, 64.1) | (74.2, 79.3) | (61.4, 61.6) | (67.7, 71.7) | (63.4, 64.1) | (61.5, 62.3) |
| 70 | I | 87.2 | 83.0 | 87.0 | 83.3 | 86.5 | 85.1 | 79.2 | 87.2 | 85.6 | 83.4 |
|  |  |  | (82.5, 83.7) | (86.8, 87.1) | (82.3, 84.4) | (86.2, 86.8) | (83.4, 87.0) | (78.9, 79.5) | (87.2, 87.3) | (85.0, 86.1) | (82.2, 84.6) |
|  | II |  | 77.4 | 84.8 | 81.2 | 84.7 | 82.5 | 76.3 | 83.9 | 81.1 | 78.8 |
|  |  |  | (76.8, 78.0) | (84.6, 85.0) | (80.0, 82.6) | (84.4, 84.9) | (80.7, 84.7) | (75.9, 76.7) | (83.3, 84.5) | (80.2, 82.0) | (77.8, 79.9) |
|  | III |  | 76.5 | 80.7 | 76.1 | 82.0 | 80.4 | 73.1 | 81.1 | 74.6 | 76.3 |
|  |  |  | (75.4, 77.7) | (80.3, 81.0) | (74.8, 77.7) | (81.8, 82.3) | (78.6, 82.5) | (72.9, 73.3) | (80.2, 82.3) | (74.4, 74.9) | (75.5, 77.2) |
|  | IV |  | 71.8 | 74.0 | 72.0 | 72.3 | 78.1 | 71.0 | 75.9 | 72.7 | 71.3 |
|  |  |  | (71.5, 72.2) | (73.7, 74.3) | (71.5, 72.7) | (72.2, 72.5) | (76.7, 79.7) | (71.0, 71.1) | (74.8, 77.3) | (72.5, 73.0) | (71.1, 71.5) |
| 80 | I | 89.9 | 87.1 | 89.8 | 86.8 | 89.0 | 87.9 | 85.3 | 89.9 | 89.2 | 86.8 |
|  |  |  | (86.8, 87.4) | (89.7, 89.9) | (85.8, 87.8) | (88.8, 89.2) | (86.6, 89.5) | (85.2, 85.5) | (89.9, 90.0) | (88.9, 89.5) | (86.2, 87.5) |
|  | II |  | 83.3 | 88.4 | 85.4 | 88.1 | 85.0 | 83.3 | 87.7 | 85.1 | 83.7 |
|  |  |  | (83.0, 83.6) | (88.3, 88.6) | (84.8, 86.1) | (88.0, 88.3) | (84.0, 86.4) | (83.1, 83.5) | (87.4, 88.0) | (84.6, 85.7) | (83.3, 84.2) |
|  | III |  | 82.5 | 86.1 | 82.2 | 85.9 | 84.4 | 81.6 | 86.5 | 82.3 | 82.7 |
|  |  |  | (82.0, 83.2) | (85.9, 86.3) | (81.7, 82.8) | (85.8, 86.1) | (83.5, 85.6) | (81.5, 81.7) | (85.9, 87.1) | (82.2, 82.5) | (82.3, 83.1) |
|  | IV |  | 80.9 | 82.3 | 80.8 | 81.1 | 83.0 | 80.7 | 83.1 | 81.3 | 80.6 |
|  |  |  | (80.8, 81.1) | (82.1, 82.4) | (80.7, 81.0) | (81.0, 81.1) | (82.3, 84.0) | (80.6, 80.7) | (82.5, 83.8) | (81.2, 81.4) | (80.5, 80.7) |

Table A 6: Stage-specific predicted age at death (95% CI) for male patients by cancer site for a range of ages at diagnosis.

| Age | Stage | General  Population | Bladder | Colorectal | Hodgkin  Lymphoma | Lung | Melanoma | Prostate | Stomach |
| --- | --- | --- | --- | --- | --- | --- | --- | --- | --- |
| 40 | I | 80.9 | 69.4 | 77.8 | 80.7 | 70.3 | 76.2 | - | 71.4 |
|  |  |  | (65.6, 73.7) | (74.9, 80.9) | (79.5, 81.9) | (67.1, 73.7) | (73.6, 79.0) |  | (64.4, 80.4) |
|  | II |  | 60.7 | 72.9 | 78.9 | 58.9 | 68.5 | - | 60.5 |
|  |  |  | (56.4, 66.0) | (71.0, 75.0) | (76.6, 81.4) | (54.6, 64.5) | (65.7, 71.6) |  | (54.8, 68.2) |
|  | III |  | 61.8 | 64.8 | 72.6 | 50.4 | 66.0 | - | 50.4 |
|  |  |  | (55.8, 70.3) | (63.2, 66.5) | (68.0, 77.9) | (48.4, 53.0) | (62.0, 70.8) |  | (47.1, 55.1) |
|  | IV |  | 45.2 | 44.5 | 73.9 | 42.5 | 54.2 | - | 45.4 |
|  |  |  | (43.0, 49.0) | (44.0, 45.2) | (71.0, 77.1) | (42.1, 43.0) | (50.6, 59.0) |  | (43.6, 48.1) |
| 50 | I | 81.7 | 74.1 | 79.2 | 81.2 | 69.9 | 79.1 | - | 72.0 |
|  |  |  | (72.5, 75.8) | (78.0, 80.5) | (79.6, 82.8) | (68.2, 71.8) | (77.8, 80.4) |  | (68.7, 76.0) |
|  | II |  | 66.7 | 75.8 | 78.7 | 63.3 | 71.6 | - | 63.5 |
|  |  |  | (64.7, 68.9) | (75.0, 76.6) | (76.4, 81.2) | (61.4, 65.6) | (70.1, 73.2) |  | (61.2, 66.2) |
|  | III |  | 66.1 | 70.2 | 72.9 | 56.3 | 70.5 | 77.7 | 59.8 |
|  |  |  | (62.7, 70.5) | (69.5, 70.9) | (69.3, 77.1) | (55.6, 57.2) | (68.2, 73.2) | (74.0, 82.0) | (58.3, 61.6) |
|  | IV |  | 54.3 | 54.2 | 74.3 | 51.6 | 60.3 | 61.4 | 52.6 |
|  |  |  | (53.2, 55.9) | (53.9, 54.5) | (71.7, 77.3) | (51.5, 51.8) | (58.2, 63.0) | (60.1, 62.8) | (52.0, 53.2) |
| 60 | I | 83.0 | 78.1 | 81.3 | 81.5 | 71.4 | 82.4 | - | 75.6 |
|  |  |  | (77.5, 78.8) | (80.9, 81.8) | (79.6, 83.5) | (70.8, 72.1) | (81.9, 82.9) |  | (73.8, 77.7) |
|  | II |  | 72.3 | 79.0 | 78.1 | 68.5 | 76.4 | - | 69.3 |
|  |  |  | (71.5, 73.1) | (78.6, 79.4) | (75.8, 80.7) | (67.9, 69.3) | (75.6, 77.3) |  | (68.1, 70.7) |
|  | III |  | 70.8 | 75.4 | 74.9 | 63.7 | 75.6 | 80.7 | 67.8 |
|  |  |  | (69.2, 72.6) | (75.0, 75.7) | (72.6, 77.7) | (63.5, 64.0) | (74.2, 77.2) | (79.6, 81.8) | (66.9, 68.8) |
|  | IV |  | 63.4 | 63.4 | 74.9 | 61.0 | 67.1 | 69.3 | 61.5 |
|  |  |  | (62.9, 64.1) | (63.2, 63.6) | (73.1, 76.9) | (61.0, 61.1) | (65.9, 68.5) | (68.9, 69.7) | (61.3, 61.8) |
| 70 | I | 85.1 | 81.8 | 84.2 | 82.1 | 76.9 | 85.1 | - | 81.5 |
|  |  |  | (81.5, 82.1) | (84.0, 84.5) | (80.0, 84.6) | (76.7, 77.2) | (85.1, 85.2) |  | (80.7, 82.4) |
|  | II |  | 77.7 | 82.7 | 79.8 | 75.2 | 81.5 | - | 76.9 |
|  |  |  | (77.4, 78.1) | (82.5, 82.9) | (78.3, 81.5) | (74.9, 75.5) | (81.1, 82.0) |  | (76.3, 77.6) |
|  | III |  | 77.0 | 80.3 | 78.6 | 72.5 | 80.0 | 84.5 | 74.9 |
|  |  |  | (76.2, 77.8) | (80.1, 80.6) | (77.2, 80.2) | (72.4, 72.6) | (79.3, 80.8) | (84.1, 84.9) | (74.4, 75.4) |
|  | IV |  | 72.3 | 72.3 | 77.5 | 70.8 | 74.5 | 77.0 | 71.1 |
|  |  |  | (72.0, 72.6) | (72.2, 72.4) | (76.4, 78.8) | (70.7, 70.8) | (73.8, 75.4) | (76.8, 77.1) | (71.0, 71.3) |
| 80 | I | 88.6 | 86.5 | 87.6 | 85.7 | 84.1 | 88.6 | - | 85.9 |
|  |  |  | (86.3, 86.6) | (87.4, 87.8) | (84.1, 88.0) | (84.0, 84.3) | (88.5, 88.6) |  | (85.5, 86.4) |
|  | II |  | 83.7 | 87.0 | 84.4 | 82.7 | 86.3 | - | 83.1 |
|  |  |  | (83.5, 83.8) | (86.9, 87.1) | (83.5, 85.5) | (82.5, 82.8) | (86.0, 86.6) |  | (82.8, 83.3) |
|  | III |  | 83.1 | 85.4 | 83.8 | 81.4 | 85.1 | 87.8 | 82.2 |
|  |  |  | (82.7, 83.4) | (85.2, 85.5) | (83.0, 84.8) | (81.3, 81.5) | (84.7, 85.7) | (87.6, 88.0) | (82.0, 82.5) |
|  | IV |  | 81.2 | 81.1 | 83.0 | 80.5 | 82.6 | 84.0 | 80.6 |
|  |  |  | (81.0, 81.3) | (81.1, 81.2) | (82.4, 83.8) | (80.5, 80.6) | (82.2, 83.2) | (83.9, 84.1) | (80.6, 80.7) |

## Appendix H


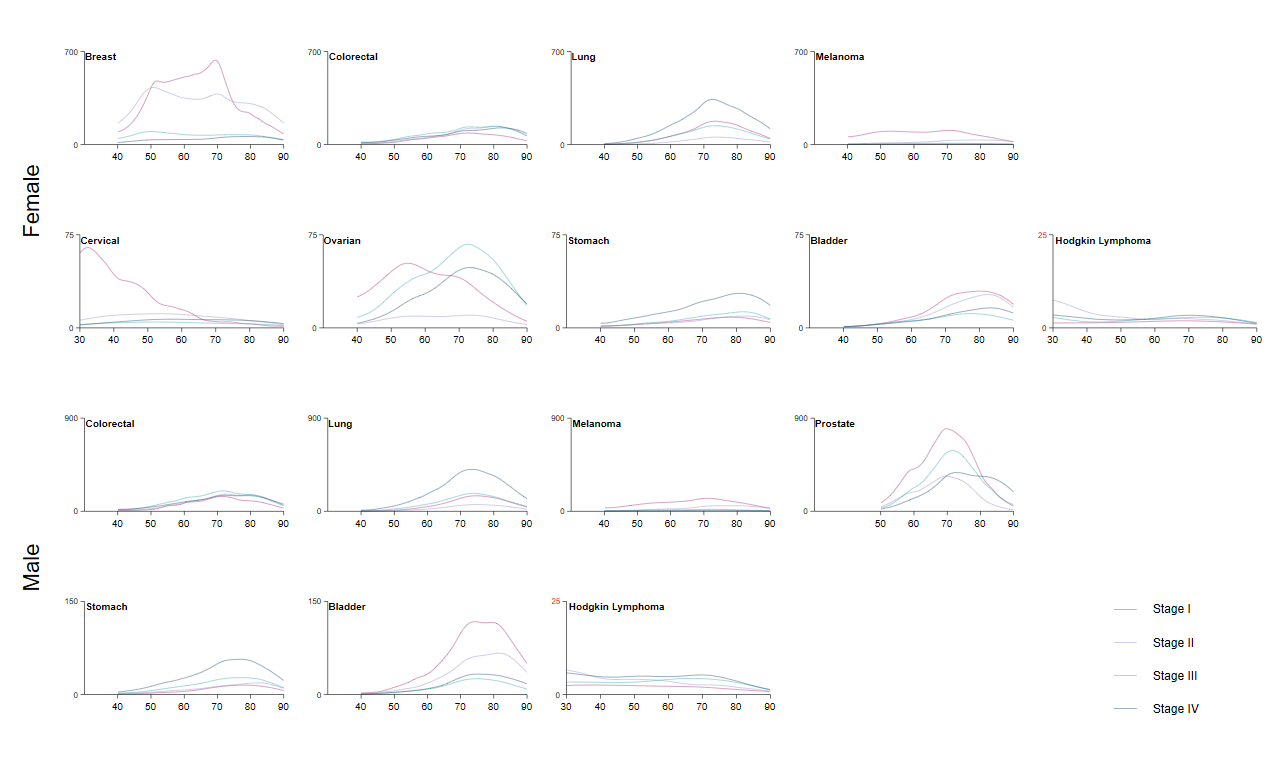


Figure A 12: Average yearly stage-specific age distribution of patients diagnosed in 2017-2019. The x-axis scale is age at diagnosis in years. The age distributions are comparable within each row as the plots are on a common y-scale, but not across rows. The y-scale for plots associated with Hodgkin Lymphoma patients only ranges from 0 to 25 due to the low incidence compared to other cancer sites.

# SUPPLEMENTARY MATERIALS

The Stata code is available at https://github.com/RStannard1/Stage-Specific-Life-Expectancy-2025.
